# The Quest for Phenolic Compounds from Macroalgae: A Review of Extraction and Identification Methodologies

**DOI:** 10.3390/biom9120847

**Published:** 2019-12-09

**Authors:** Sónia A. O. Santos, Rafael Félix, Adriana C. S. Pais, Sílvia M. Rocha, Armando J. D. Silvestre

**Affiliations:** 1 CICECO—Aveiro Institute of Materials, Department of Chemistry, University of Aveiro, 3810-193 Aveiro, Portugal; a.c.p.s@ua.pt (A.C.S.P.); armsil@ua.pt (A.J.D.S.); 2 On Leave MARE—Marine and Environmental Sciences Centre, ESTM, Instituto Politécnico de Leiria, 2520-620 Peniche, Portugal; rafaelfariafelix95@gmail.com; 3 QOPNA/LAQV-REQUIMTE, Department of Chemistry, University of Aveiro, 3810-193 Aveiro, Portugal; smrocha@ua.pt

**Keywords:** bioactive compounds, extraction, characterization, macroalgae, phenolic compounds, phlorotannins, mass spectrometry, seaweed

## Abstract

The current interest of the scientific community for the exploitation of high-value compounds from macroalgae is related to the increasing knowledge of their biological activities and health benefits. Macroalgae phenolic compounds, particularly phlorotannins, have gained particular attention due to their specific bioactivities, including antioxidant, antiproliferative, or antidiabetic. Notwithstanding, the characterization of macroalgae phenolic compounds is a multi-step task, with high challenges associated with their isolation and characterization, due to the highly complex and polysaccharide-rich matrix of macroalgae. Therefore, this fraction is far from being fully explored. In fact, a critical revision of the extraction and characterization methodologies already used in the analysis of phenolic compounds from macroalgae is lacking in the literature, and it is of uttermost importance to compile validated methodologies and discourage misleading practices. The aim of this review is to discuss the state-of-the-art of phenolic compounds already identified in green, red, and brown macroalgae, reviewing their structural classification, as well as critically discussing extraction methodologies, chromatographic separation techniques, and the analytical strategies for their characterization, including information about structural identification techniques and key spectroscopic profiles. For the first time, mass spectrometry data of phlorotannins, a chemical family quite exclusive of macroalgae, is compiled and discussed.

## 1. Introduction

**Figure 1:**
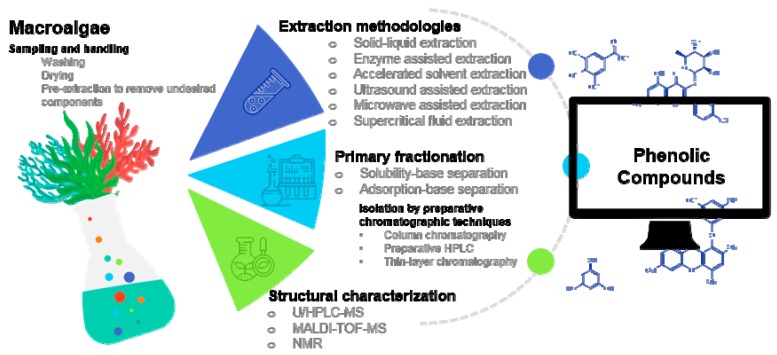
Schematic representation of the multi-step strategy applied to the identification of macroalgae phenolic compounds.

Phenolic compounds constitute one of the most numerous and widespread groups of secondary metabolites. These components are important to the normal growth and development of algae and terrestrial plants, providing defense mechanisms against infections, injuries, and environmental aggressions. As part of both animal and human diet, the nutraceutical properties assigned to phenolic compounds are almost endless [1]. Therefore, in recent years, there has been an outstanding demand for the search for phenolic compounds from natural sources, with a focus on plants [2], fruits [3], or ensuing agro-industrial biomass residues [4]. Marine macroalgae (seaweeds) have been seen in the last years as a valuable source of bioactive components, including phenolic compounds [5], which in some cases are quite exclusive to macroalgae, e.g., phlorotannins. Despite the growing number of studies regarding the analysis of the phenolic fraction of macroalgae [6,7], including the optimization of extraction conditions [8,9] and analysis [10] of such components, a detailed and comprehensive revision of their phenolic composition as well as of the extraction and analytical methodologies applied to their detailed characterization, has not been available so far. Actually, and despite the increasing interest from the scientific community, the bioprospection of phenolic compounds from macroalgae has been a challenging issue. The large size and complexity of their structures, together with the high abundance of polysaccharides on the macroalgae matrix, make the isolation and characterization of phenolic fraction quite difficult. On this vein, a multi-step strategy (Figure 1), including different fractionation techniques after phenolic compounds extraction, has been done by different scientists, which influenced afterward the effectiveness of the structural characterization.

Therefore, the aim of this review is to provide a comprehensive overview of published information on the phenolic composition of green, red, and brown macroalgae, reviewing their structural classification, as well as critically discuss extraction methodologies, chromatographic separation methodologies, and structural identification techniques and key spectroscopic profiles of identified phenolic compounds. Due to the well-known significant inter- or either intra-species variability of macroalgae composition, depending on both macroalgae characteristics (e.g., age, size, tissue type) and abiotic factors (e.g., nutrients, salinity, light, season), phenolic content values will not be discussed in detail here. Most studies here revised have reported total phenolic (TPC) or phlorotannins contents (TPhC) only based on spectrophotometric methods, as will be discussed below, for which the possible co-extraction of, e.g., polysaccharides may have a significant contribution.

This appraisal can constitute a landmark on the systematization of the multi-step procedures used to study macroalgae, fostering research and the valorization of a very abundant source of natural compounds, representing, therefore, an important contribution to the development of the so-called blue biotechnology, one of the most important areas for the sustainable world development.

## 2. Wide Chemical Diversity of Phenolic Compounds Present in Macroalgae

Structurally, phenolic compounds present in macroalgae vary from simple molecules, such as phenolic and cinnamic acids or flavonoids, to the more complex phlorotannin polymeric structures.

### 2.1. Simple Phenolic Compounds

**Figure 2:**
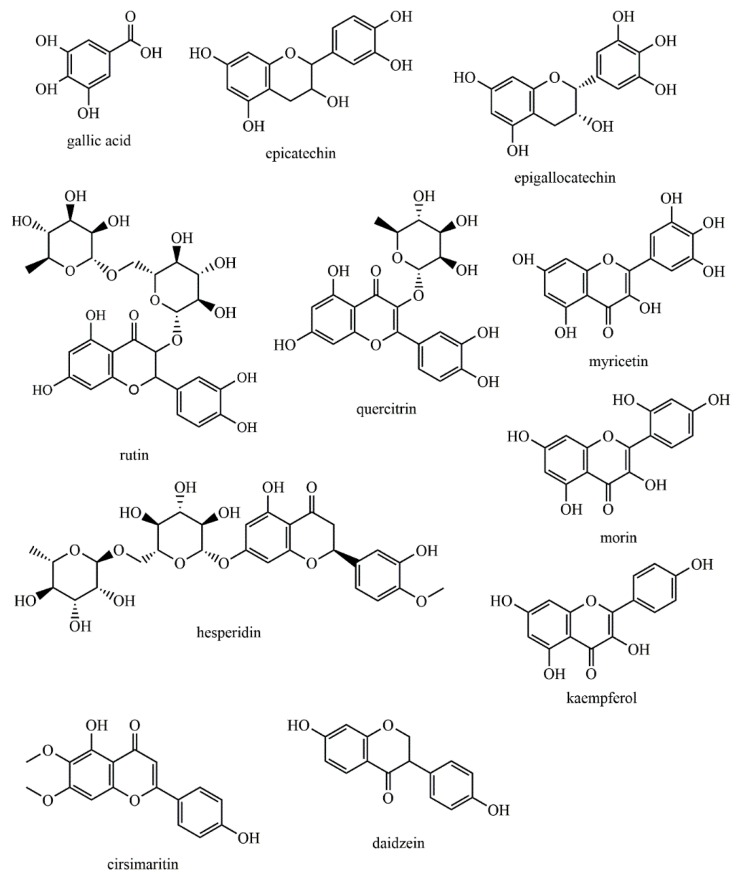
Major phenolic compounds identified in green, red, and brown macroalgae.

Hydroxybenzoic acid derivatives, such as gallic acid (Figure 2), are commonly reported as constituents of different green, red, and brown macroalgae species [11,12,13,14]. Flavan-3-ol derivatives, such as epicatechin or epigallocatechin (Figure 2), have been one of the major class of phenolic components detected in green, red, and brown macroalgae [11,15]. Other flavonoids, such as rutin, quercitrin, hesperidin, myricetin, morin, kaempferol, and cirsimaritin (Figure 2), were detected in several Chlorophyta, Rhodophyta and Phaeophyta species [14,16,17]. Compounds restricted to specific macroalgae species have also been observed. Different isoflavones, such as daidzein or genistein (Figure 2), have been identified in the red macroalgae *Chondrus crispus*, *Halopytis incurvus*, and *Porphyra* sp. and in the brown ones *Sargassum muticum*, *Sargassum vulgare*, and *Undaria pinnatifida* [18], while a considerably high number of flavonoid glycosides have been found in the brown macroalgae *Durvillaea antarctica*, *Lessonia spicata*, and *Macrocystis integrifolia* [19]. Coumarins have also been identified in the brown macroalga *Padina tetrastromatica* [20] and in the green macroalga *Dasycladus vernicularis* [21]. Halogenated derivatives of phenolic compounds have also been reported as constituents of macroalgae, including simple structures, such as brominated derivatives of hydroxybenzoic acids, already detected in the green macroalgae *Ulva lactuca* [22], or more complex classes, such as the two chlorinated aurones reported in the brown one *Spatoglossum variabile* [23]. Additionally, a vast number of sulphated phenolic compounds have already been detected in several Chlorophyta, Rhodophyta and Phaeophyta species [21,24,25].

Uncharacteristic components have also been detected in some macroalgae, such as carnosic acid, a phenolic abietane based diterpene commonly found in flowering plants [26], which has already been detected in *Himanthalia elongata* [17].

### 2.2. Phlorotannins

**Figure 3:**
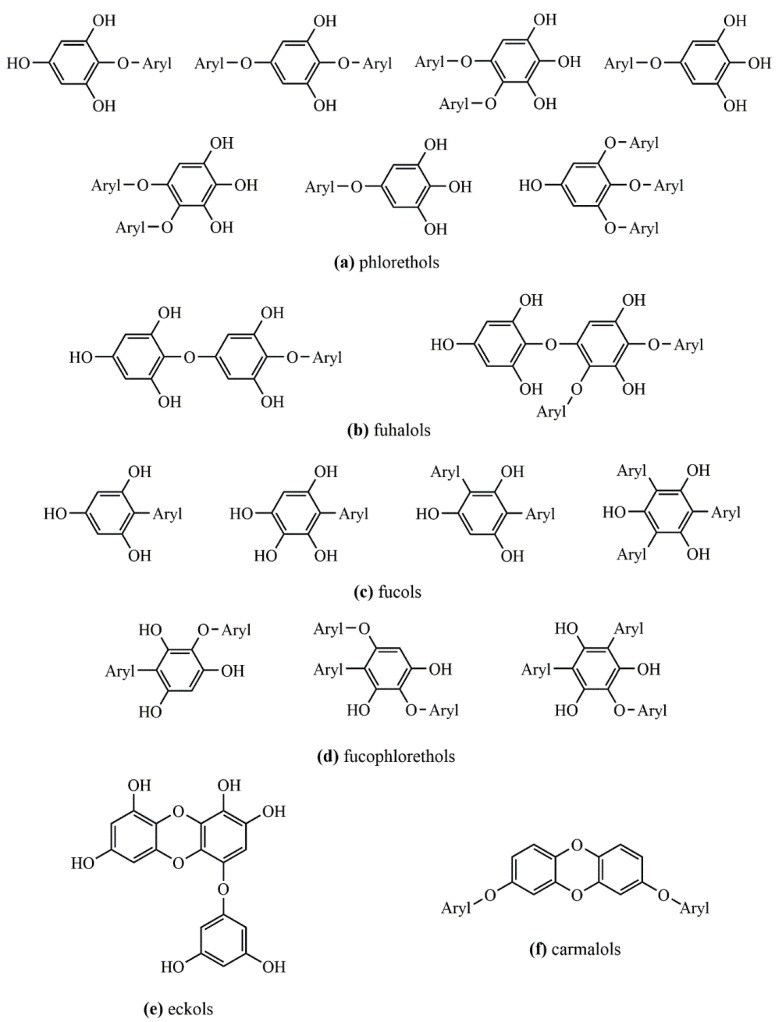
Generic structure of phlorethols (**a**), fuhalols (**b**), fucols (**c**), fucophlorethols (**d**), eckols (**e**), and carmalols (**f**). Adapted from [31].

The often-recognized high content of phenolic compounds in brown macroalgae (as compared to green and red ones), is normally associated with phlorotannins, a restricted class of polyphenols derived from the oligomerization of phloroglucinol (1,3,5-trihydroxybenzene) units (PGU) through diaryl ether or C–C bonds. This class comprises compounds with a large range of molecular sizes, ranging from 126 Da (phloroglucinol) to several kDa. There is no consensus about the largest molecular size of phlorotannins. Several authors reported values as high as 100 [27,28] or 650 kDa [29], despite the fact that no evidence of such values has been confirmed. Phlorotannins can be classified into four groups depending on the type of linkage between aromatic units, namely, phlorethols and fuhalols, possessing an ether linkage; fucols, possessing a phenyl linkage; fucophlorethols, possessing an ether and a phenyl linkage; and eckols and carmalols, possessing a benzodioxin linkage [30,31], as depicted in Figure 3.

**Figure 4:**
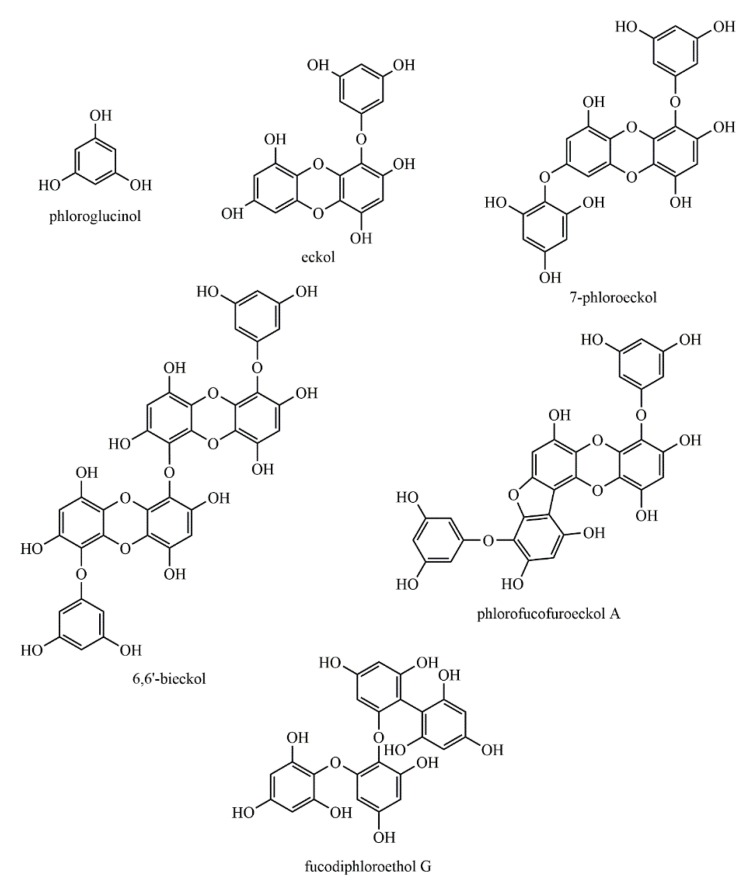
Major phlorotannins identified in brown macroalgae.

Although some species, such as those from the *Ecklonia* [32,33] and *Fucus* genera [10,28,34], are known to contain significantly higher contents of phlorotannins; these components have been identified in a vast number of other macroalgae species [19,35,36,37,38,39,40,41,42,43], with quite variable profiles and abundances. Phloroglucinol, eckol, 7-phloroeckol, 6,6′-bieckol, phlorofucofuroeckol A, and fucodiphloroethol (Figure 4) have been the most frequently reported phlorotannins in brown macroalgae [32,33,35,38,43]. In fact, the majority of the published studies have reported phlorotannins with a degree of polymerization (DP) below 10 PGU [32,33,35,37,43]. However, the availability, in the last years, of more advanced and powerful separation and structural characterization techniques have allowed the discovery of phlorotannin structures with higher numbers of repeating units. As examples, Heffernan et al. detected phlorotannins with up to 16 units of phloroglucinol in four brown macroalgae (*Fucus serratus*, *Fucus vesiculosus*, *Himanthalia elongata*, and *Cystoseira nodicaulis*) from the Irish coast [28], while phlorotannins containing up to 17 and 27 PGU were also identified in *Cystoseira abies-marina* [36] and *Laminaria digitata* [44], respectively. Finally, the largest phlorotannins characterized so far, with 49 PGU, have been detected in *Fucus vesiculosus* [10].

Finally, halogenated and sulphated derivatives of phlorotannins are also commonly present in brown macroalgae species [39,40,41,45,46,47]. Bromo, iodo, and chloro derivatives of phlorotannins are commonly composed of up to 3 PGU, being mainly derivatives of phloroglucinol, phlorethols, and eckols [41,45,46], although chlorobifuhalol and chlorodifucol have been already detected in *Carpophyllum angustifolium* [40]. A sulphated bromophloroglucinol was also identified in *Polysiphonia lanosa* [47], while 8 sulphated phlorotannins with 1 or 2 PGU, namely phloroglucinol and difucol or diphlorethol derivatives, were detected in *Pleurophycus gardneri* [39].

### 2.3. Biological Activities of Macroalgae Phenolic Compounds

Phenolic compounds derived from macroalgae are already known for their numerous biological activities, which have been summarized in many studies [29,48,49]. In the myriad of biological activities addressed to phenolic compounds obtained from macroalgae are included anti-alzheimer, anti-inflammatory, anti-allergic, anti-proliferative, antioxidant, anti-obesity, bactericidal activities, among others [50]. In particular, phlorotannins, which are present in abundance in brown macroalgae, have also been identified as potential health promoters and related to a reduction of diseases risk [29].

Dieckol, eckol, fucodiphlorethol G, and phloroglucinol are some of the phlorotannins isolated from *Ecklonia cava*, which present antioxidant activity [49]. Moreover, some phlorotannins obtained from this macroalga, namely fucodiphloroethol, dieckol, eckol, and phlorofucofuroeckol, have shown a potent cytotoxic effect on human cancer cell lines (i.e., HeLa, HT1080, A549, among others) [29]. The bactericidal activity of dieckol from *Ecklonia kurome* has already been evidenced against some food-borne pathogenic bacteria, since this phlorotannin reduces the growth of *Campylobacter jejuni* and *Vibro parahaemolyticus* [29]. Furthermore, the anti-inflammatory activity of phlorofucofuroeckol-A was showed in LPS-induced RAW264.7 cells [49].

Despite the vast range of biological activities already addressed to macroalgae phenolic compounds, their exploitation in cosmetic, nutraceutical, or pharmaceutical applications requires, on one hand, the development of expedite green and sustainable extraction methodologies and, on the other hand, the use of successful tools for extracts, fractions, or individual compound characterization [49].

## 3. Extraction of Phenolic Compounds from Macroalgae

The extraction of phenolic compounds from natural sources is a complex task due to the effect of different parameters such as the sample particle size, the extraction method employed, the extraction time and temperature, the storage conditions (for both raw material and extracts), or the presence of interfering components [51]. Additionally, the sampling and extraction conditions must be carefully selected in order to avoid possible degradation of the phenolic compounds. Temperature, oxygen, and light exposures are the main factors promoting the degradation reactions of phenolic compounds [52,53].

### 3.1. Sampling Procedures and Sample Handling before Phenolic Compounds Extraction

Macroalgae are generally harvested from coastal areas, beaches, or aquaculture, being then washed with seawater, preferably followed by tap water [14,42,54,55,56,57,58,59,60], to remove salt residues, epiphytes, or other impurities. Although in some studies macroalgae have been submitted to extraction immediately after washing [61], in most cases, and in order to stop ongoing biochemical processes, macroalgae samples are commonly deep-frozen [62,63,64] to be used as fresh material, or dried before extraction [14,19,44,58,59,65]. Drying techniques can considerably affect the extraction efficiency of phenolic compounds from macroalgae. For example, the oven-drying at 40 °C of the red macroalgae *Kappaphycus alvarezii* allowed obatining extracts with higher TPC, total flavonoids content (TFC), and total anthocyanin content (TAC) as well as higher antioxidant activity when compared with other six drying techniques [66]. The same drying temperature (in a studied range between 25 and 40 °C) was also reported to give the highest TPC of *Himanthalia elongata* extracts [67]. Notwithstanding, Cruces et al. verified that the freeze-drying of macroalgae samples enhance the TPhC of *Lessonia spicata* extracts when compared with other techniques such as freezing, silica-drying, oven-drying, or air drying [68].

Commonly, to avoid the co-extraction of undesired components (such as lipidic components and/or pigments), the extraction of phenolic components from macroalgae is frequently preceded by a pre-extraction with a less polar solvent, such as n-hexane [34,35,56,69,70,71], n-hexane:acetone [72], n-hexane:ethyl acetate mixtures [73], or dichloromethane [21]. These solvents or mixtures have shown to be particularly efficient in extracting macroalgae lipophilic compounds [74,75]. As an alternative to solvent extraction, De Corato et al. treated different dried brown and red macroalgae with sodium hydroxide (1:10, *w/w*) for 24 h for lipids saponification before phenolic compounds, particularly phlorotannins, extraction [42]. Finally, some authors have removed undesired components only after the extraction of the phenolic compounds by liquid–liquid partitioning with dichloromethane [10], petroleum ether [62], or a sequence of solvents [76], which will be later discussed in Section 5.1.

### 3.2. Solvent Selection

The choice of the extraction solvent or solvent mixtures is one of the main concerns for the success of any extraction process. Organic solvents are still the most successfully applied in the extraction of phenolic compounds from macroalgae. Binary aqueous mixtures with methanol, ethanol, acetone, or acetonitrile have been most often used [10,17,19,33,35,44,58,77]. Although there is no consensus on the best solvent/mixture of solvents to be used, several authors have reported better extraction efficiencies with organic aqueous mixtures than the ones obtained with a single solvent [14,77,78], with the exception of Leyton et al. who observed a higher TPC in the brown macroalgae *Macrocystis pyrifera* extracts using water as solvent when compared with different organic solvents or organic aqueous mixtures [69]. Even so, an increase in the polysaccharides extraction should not be discarded to contribute to higher TPC. Notwithstanding, Nwosu et al. verified a noteworthy increase in the TPC of different extracts from edible macroalgae when these were prepared with acetonitrile:water (50:50, *v/v*) instead of with methanol [77]. A higher TPC of different brown macroalgae extracts was also verified when an ethanol:water mixture (80:20, *v/v*) was used for the extraction instead of water (even at high temperatures) [78]. Additionally, the effect of ethanol percentage in water on the phlorotannins extraction of *Eisenia bicyclis* extracts was studied by Kim et al., which verified that a maximum on both extraction yield and phlorotannins content, quantified by high-performance liquid chromatography (HPLC), was reached when 50% aqueous ethanol was used for the extraction [38]. The extraction of *Fucus vesiculosus* with acetone:water (70:30, *v/v*) obtained a higher content of soluble phlorotannins than with other solvents or mixtures [79,80]; this is one of the most frequently used extraction solvent mixtures in the studies regarding phenolic compounds from macroalgae [35,60,70]. Catarino et al. optimized the acetone concentration (in a range from 30% to 70% (*v/v*)) in order to maximize the TPhC of *Fucus vesiculosus* acetone:water extracts, reaching a maximum at 67% of acetone [9]. Additionally, in a scale-up perspective, the benign character of acetone has been up and coming in the increasing demand in the last years to achieve efficient extraction methodologies with a reduction, or an absence, of unsafe organic solvents consumption. In fact, several alternative solvents, such as ionic liquids (IL) and deep eutectic solvents (DES), given their tunable properties, have emerged with remarkable potential for the extraction of added value compounds [81,82,83]. Notwithstanding, none of these solvents have been so far been applied to the extraction of phenolic compounds from macroalgae, although other novel and sustainable extraction methodologies have been exploited in the extraction of phenolic compounds from macroalgae, as will be discussed in Section 3.3.2.

The addition of antioxidant agents to the extraction media has been reported to increase the stability of phenolic compounds in general and thus to preserve their antioxidant potential [84] and inevitably of those present in macroalgae, particularly phlorotannins. Ascorbic acid has already been reported to be added to macroalgae extracts before analysis in order to prevent phlorotannin oxidation [18]. The use of up to 0.3% of ascorbic acid (*w/v*) has been shown to enhance both the number and quantity of phlorotannins in *Fucus vesiculosus* extracts [34]. Finally, in some studies, phlorotannins rich extracts have been further derivatized through acetylation in order to prevent oxidation [41,43,62].

### 3.3. Extraction Methodologies

Similar to other botanical sources, solid–liquid extraction (SLE) is still the most common procedure to extract the phenolic fraction from macroalgae, due to its simplicity, efficiency, and easy tunning as well as scalability. However, over the last years, more efficient and/or environmentally friendly extraction methods have been studied.

#### 3.3.1. Conventional Solid–Liquid Extraction

Conventional SLE, also called maceration, in which the components are removed from a plant-based matrix by submerging the matrix in an appropriate solvent/solvent mixture, is the most traditional technique used to extract phenolic compounds [85], being also the most frequently applied in macroalgae [38,77,86]. Apart from the solvent used (discussed above), the main parameters affecting the efficiency of this technique are the temperature, extraction time, and solid/liquid ratio (SLr). Different conventional SLE approaches employed for the isolation of phenolic compounds fractions from macroalgae are summarized in Table 1. Most of the reported studies have performed the extractions at room temperature [10,12,28,34,35,38,59,66,70,77,78,79,80,87,88]. Actually, the use of higher extraction temperatures has been described to promote the oxidation of phenolic compounds [53]. Catarino et al. demonstrated that the highest TPhC of *Fucus vesiculosus* extracts is obtained at 25 °C, in a studied range between 15 and 50 °C [9]. Notwithstanding, other authors have verified a positive effect of the extraction temperature on the phlorotannins recovery, namely in *Eisenia bicyclis* [38] and *Macrocystis pyrifera* [69], for which the best temperatures were reported to be 80 and 55 °C, respectively. However, these different findings may be related to the inter-species variability of phlorotannin profiles, and thus on different sensitivities to temperature.

A wide range of extraction times have been reported in the literature for the extraction of phenolic compounds from macroalgae, with values as short as 5 min [86] or as long as 30 days [61] being found. However, 24 h has been amongst the most frequently used extraction time [59,78,80,89,90].

More consensus is observed in the SLr used, with most authors employing ratios (*w/v*) of 1:10 [28,77,89] or 1:20 [59,70,78]. Several authors have already studied the effect of the SLr on phlorotannin content. The optimal SLr determined for the phlorotannins extraction from *Macrocystis pyrifera* was reported to be 1:15 [69] (within a studied range between 1:10 and 1:20), while, according to other authors, no SLr effect (in the range of 1:2 and 1:10) was observed on the phlorotannin content of *Eisenia bicyclis* [38]. A different SLr effect was observed on the phlorotannin extraction from *Fucus vesiculosus*, for which 1:70 was the optimal value (in a range between 1:10 and 1:110) [9]. Finally, the conventional SLE of phenolic compounds from macroalgae has also been commonly assisted by stirring [9,28,62,69,86,88,91] in order to increase molecular diffusion.

Although unusual in the extraction of phenolic compounds due to the higher temperatures of the extraction involved, at least two studies have used the Soxhlet apparatus in the extraction of phenolic compounds from brown macroalgae, namely from the macroalga *Sargassum wightii* [56], from which some phlorotannins were identified after fractionation, and from *Padina pavonica* [92]. In this last study, the phenolic fraction was only characterized by spectrophotometric methods, namely, in terms of TPC, TFC, and total tannins content (TTC) (see Section 4.1).

Despite its simplicity, easy adjustment, and efficiency that makes conventional SLE the first approach in studying the phenolic composition of macroalgae, its limitations (namely, being time-consuming, the large volumes of organic solvents (often unsafe) required and, therefore, high cost from an industrial application perspective) have meant that more efficient, environmentally friendly, and sustainable techniques have been pursued in recent years.

#### 3.3.2. Novel Extraction Methodologies

Algae cell walls are composed of fibrous composites of microfibrillar polysaccharides embedded in matrix polysaccharides and proteoglycans [93]. These components are a physical obstruction to the normal release of bioactive components, such as phenolic compounds. Additionally, other aspects can hamper their extraction, such as the gelling properties of some polysaccharides, namely, alginate and laminarin-like ones, or the strong complexes that phlorotannins can form with proteins by either non-covalent or covalent bonds [94]. Therefore, and despite the environmental and sustainability concerns, a number of novel and alternative extraction methodologies have been already investigated in the extraction of phenolic compounds from macroalgae, namely, enzyme assisted extraction (EAE), accelerated solvent extraction (ASE), ultrasound assisted extraction (UAE), microwave assisted extraction (MAE), and supercritical fluid extraction (SFE).

##### Enzyme Assisted Extraction

Several authors have applied EAE in the extraction of phenolic compounds from different green, red, and brown macroalgae species [95,96,97]. Therefore, the use of enzymes to break down all these macromolecules seems to be an important step to improve the extraction of phenolic compounds. Different enzymes and EAE conditions have been used for this, as summarized in Table 2. Carbohydrases and proteases have been the most commonly used enzymes, either isolated [98], combined [71,99], or even using multi-enzyme complexes [95,96,97,100]. Some authors have combined the enzymatic treatments with other extraction methodologies, such as conventional SLE [99], MAE [100], or ASE [96]; however, most studies (Table 2) only used the aqueous medium as extraction solvent at the optimum conditions of the different enzymes used, which certainly limits the extraction of the target compounds.

In general, the macroalgae extracts obtained by EAE have TPC similar to those obtained by conventional SLE with water [95,99], although Wang et al. verified an increase in the TPC of green, red, and brown macroalgae extracts obtained by EAE, especially using proteases, when compared with conventional water extraction [97]. Nevertheless, Olivares-Molina and Férnandez observed a significant decrease on the TPC of *Lessonia nigrescens*, *Macrocystis pyrifera*, and *Durvillaea antarctica* extracts obtained by EAE when compared to those obtained by conventional SLE using acetone:water as extraction solvent [98]. In fact, the lower TPC is certainly related with the aqueous medium used in the EAE, and the lower affinity of macroalgae phenolic compounds for this solvent, as aforementioned.

In order to increase the phenolic compounds extraction yields from macroalgae, some authors have studied the combination of EAE with other techniques. Charoensiddhi et al. obtained *Ecklonia radiata* extracts with higher TPC and antioxidant activity using a microwave-assisted enzymatic extraction than with conventional SLE or single EAE [100]; Siriwardhana et al. verified an increase in the TPC and antioxidant activity of brown macroalga *Hizikia fusiformis* extracts when EAE is combined with conventional SLE [99]; while Sánchez-Camargo et al. studied the effect of applying ASE (methodologies discussed in more detail below) in the biomass residue obtained after EAE [96]. This sequential methodology led to macroalgae extracts with lower TPC than when ASE was applied to non-hydrolysate macroalgae. However, this should certainly be related to components that were extracted during EAE and discarded, not being accounted for in the TPC determination. Finally, it should be highlighted that only a limited number of studies regarding the use of EAE in the extraction of the phenolic compounds from macroalgae have characterized in detail the final extracts obtained [71,95,96], with the vast majority just evaluating the phenolic content by spectrophotometric assays.

##### Accelerated Solvent Extraction

ASE, also known as pressurized liquid extraction (PLE), is another promising process for the phenolic compounds extraction, providing shorter extraction time, reduced solvents consumption, and higher extraction efficiency. This technique uses solvents at high pressures and temperatures, accelerating the extraction process. The high temperatures used increase solubility, the extraction kinetics, and, at the same time, decrease the solvent viscosity, enhancing diffusion and sample penetration, facilitating the target components’ desorption [103]. Despite the high temperatures used, it is claimed that in ASE, the degradation of phenolic compounds does not occur due to the absence of air and light [53], being one of the main advantages of this technique.

A number of studies have already applied ASE in the extraction of phenolic compounds from macroalgae [8,90,96,104,105,106]. Ethanol:water mixtures have been the most frequently used extraction solvents, as shown in Table 3. The pressures used ranged from 1000 [90] to 1500 psi [8,96,105], with (static) extraction times ranging from 5 [106] to 25 min [90], although most studies have performed ASE for 20 min [8,96]. Sánchez-Camargo et al. optimized the ethanol percentage (95%) and temperature (160 °C) for the ASE of phenolic compounds, namely phlorotannins, from the brown macroalga *Sargassum muticum* [96]. These conditions were then applied by the same authors [105] to study the geographical variability of phlorotannins composition on this species. *Sargassum muticum* was also the macroalga used by Anaëlle et al. to compare different novel techniques in the extraction of phenolic compounds from macroalgae, verifying a higher TPC in the extracts obtained by ASE with ethanol:water (75:25, *v/v*) [8]. Different solvent mixtures, temperatures, and pressures were applied in the ASE of phenolic compounds from *Fucus serratus*, *Laminaria digitata*, *Gracilaria gracilis*, and *Codium fragile* [90]; however, in this case, the authors reported that the extracts obtained presented lower TPC than those obtained by conventional SLE with the same solvent mixtures. This indicates that more in depth studies regarding the optimization of ASE of phenolic compounds from macroalgae are needed.

##### Microwave-Assisted Extraction

MAE is an SLE technique in which the system is heated by microwave irradiation. The energy transfer occurs via both dipole rotation and ionic conduction mechanisms, leading to a local and very rapid heating of the sample. During microwave heating, the dipole rotation causes disruption of the weak hydrogen bonds, as at the same time, the migration of dissolved ions occurs, increasing the penetration of the solvent into the matrix. Additionally, there is a move up of the pressure inside the matrix, which increases its porosity, also enhancing the solvent penetration [107]. Furthermore, since it is often carried out in closed vessels, MAE allows working at higher temperatures than those achievable at atmospheric pressure in SLE. All these features impart MAE with some advantages such as short extraction time, lower SLr, and, consequently, lower solvent volume consumption.

Table 4 summarizes the studies concerning the MAE extraction of phenolic compounds from macroalgae. Ethanol:water mixtures have been the most commonly used in MAE of phenolic compounds from macroalgae [108,109], although few authors have applied acetone:water mixtures [54], methanol:water [110], or just water [111]. Pérez et al. studied and optimized the MAE of phenolic compounds from *Sargassum muticum* wet samples in two stages, with a solvent-free extraction followed by a water extraction, adding only the moisture content [112]. Additionally, this approach was used in sequence with other methodologies, namely, following EAE or followed by autohydrolysis. In all the approaches, both TPC and antioxidant activity were higher than those obtained with conventional SLE tested at the corresponding conditions. Finally, MAE using ethanol or ethanol:water (50:50) were performed, yielding *Sargassum muticum* extracts with much lower TPC; however, no optimization of these approaches was performed. He et al. verified that an ethanol concentration of 55% (from a studied range between 50% and 70%) maximized the TPC of *Saccharina japonica* extracts, while other parameters, such as SLr (1:8), temperature (60 °C), irradiation power (400 w) and time (25 min) were also optimized [108]. 

A similar ethanol percentage (60%) was also verified to maximize the TPC and antioxidant activity of extracts obtained from the MAE of the green alga *Caulerpa racemosa* [109]. Other parameters, namely SLr (1:40), extraction time (40 min), irradiation power (200 w) and temperature (50 °C) were also optimized in this study. The SLr used in MAE varied from low values, such as 1:3 [111] or 1:4 [108], to considerable higher ratios, such as 1:50 [109]. In a previous study it was verified that the TPC of *Caulerpa racemosa* MAE extracts increased more than fivefold when the SLr increased from 1:10 to 1:20, after which the increasing trend remains almost insignificant [109]. Temperature seems to have also an important effect in the TPC of the macroalgae MAE extracts, with several authors reporting a decrease in the TPC at higher temperatures. Michalak et al. verified a considerable decrease in the TPC of red and green macroalgae extracts when increasing the MAE temperature from 25 to 40 or 60 °C [111]. He et al. also reported a higher TPC for the brown macroalga *Saccharina japonica* MAE extracts at 60 °C, from a studied range between 45 and 65 °C [108], while Li et al. observed an increase in the TPC of the green alga *Caulerpa racemosa* MAE extracts in a temperature range between 20 and 40 °C [109], followed by a considerable decrease at higher temperatures. Although these findings have been based only on spectrophotometric assays, they might be related also with the different composition of macroalgae species on thermally labile components, which indicates that temperature may have a significant effect on most MAE of macroalgae phenolic compounds and should, therefore, be studied in future works involving this technique.

Irradiation power and extraction time have also been optimized by several authors. Extraction times between 25 [108] and 40 min [109] have been shown to maximize the TPC of different MAE macroalgae extracts. Notwithstanding, Safari et al. verified that operating the MAE at 300 w for 8 min maximized both TPC and antioxidant activity of *Chaetomorpha sp*. MAE extracts [54]. Actually, the use of higher irradiation power values also had a negative effect on the TPC of *Caulerpa racemosa* MAE extracts [109], and Pérez et al. also observed a maximum on the TPC of *Sargassum muticum* MAE extracts using a irradiation power of 200 W (from a studied range between 200 and 900 W) [112]. Microwave irradiation was also used to assist EAE of *Ecklonia radiata*, allowing obtaining extracts with higher TPC and antioxidant activities than those obtained with conventional SLE or with EAE at the same conditions [100].

Again, most studies concerning the MAE of phenolic compounds from macroalgae have not characterized the extracts obtained, revealing a major weakness in these works. However, a recent study showed that this technique is able to extract different families of phenolic compounds, including phenolic acids, flavonoids, and phlorotannins from different brown macroalgae species [110], which point out that MAE may be a promising technique when the whole phenolic fraction of macroalgae is intended for study.

##### Ultrasound-Assisted Extraction

UAE is a non-thermal extraction method in which the solid matrix is immersed in a solvent and submitted to ultrasound irradiation by using an ultrasound bath or probe. It has become an emerging technique in the extraction of phenolic compounds. UAE uses sound waves at frequencies over the human hearing values (20 kHz), which propagate by rarefactions and compression, creating vapor bubbles. These undergo implosive collapse, known as cavitation, producing physical, chemical, and mechanical effects, which results in the disruption of the biological membranes, thus enhancing the release of the target compounds [113]. The non-thermal nature of UAE is expected to circumvent the limitations associated with other extraction techniques such as conventional SLE, MAE, and ASE in which temperature plays a key role in the global extraction efficiency, but that simultaneously has a deleterious effect over the phenolic composition, as mentioned above.

Some authors have already successfully applied UAE in the extraction of phenolic compounds from macroalgae. Table 5 summarizes the conditions employed in those studies. The ultrasound frequencies used (when reported) ranged from 20 [55,114] to 60 kHz [95], with the extraction time values ranging between 5 [55] and 90 min [115], although an UAE of 2 days has already been applied to extract phlorotannins [116]. It must be highlighted that most authors using UAE have identified the phenolic compounds extracted, contrary to most of the other novel extraction techniques. 

In addition, a high number of phenolic components were extracted when water or methanol:water mixtures were used [21,58,116,118]. These were also the most frequently used solvents in the UAE of macroalgae phenolic compounds.

Some authors reported the effect of different UAE conditions on the phenolic compounds extraction. Kadam et al. observed that an increase in the ultrasonic amplitude (from 22.7 to 114 µm) as well as that the use of diluted aqueous solution of HCl (0.03 M) instead of water in the UAE of phenolic compounds from *Ascophyllum nodosum* leaded to a considerable increase in the TPC of the extracts [114]. Later, the same authors [55] optimized the extraction time (25 min), HCl concentration (0.03 M), and ultrasonic amplitude (114 µm) of this UAE for maximum TPC by surface response methodology. Although several phlorotannins have been detected, the UAE optimization was based only on a spectrophotometric assay (see Section 4), which might compromise the real optimal conditions for phenolic compounds extraction. Actually, a study by Topuz et al. has pointed out the differences between the optimal UAE conditions to maximize TPC (SLr 1:30, *w:v*, temperature 50 °C, and extraction time 42.8 min) and those to maximize antioxidant activity (SLr 1:24.3, *w:v*, temperature 45.3 °C, and extraction time 58 min) [117], which will be more related with the presence of phenolic compounds. Notwithstanding, the efficiency of the UAE to extract phenolic compounds from macroalgae, particularly phlorotannins, has been demonstrated by the high number of components detected in such extracts (mostly after further fractionation) [58,116,118]. Additionally, it was already reported that the UAE obtained higher molecular weight phlorotannins (4-12 PGU) from *Ascophylum nodosum* than those obtained by conventional SLE (4-7 PGU) [55].

UAE has been also applied combined with conventional maceration [58,95,118,119]. The use of this technology as a pretreatment of different macroalgae samples before the SFE of isoflavones was also accessed [18]. The sonication of the macroalgae with the SFE modifier mixture (methanol:water, 10:90/*v:v*) for 30 min, by both sonication bath or thorn sonication, showed to be crucial for the SFE of the isoflavones fraction, probably due to the damage of the cell walls or organelles of the matrix prior to the SFE, enhancing the mass transfer.

##### Supercritical Fluid Extraction

SFE is becoming an attractive alternative method for the extraction of high valuable compounds from natural sources [121,122]. A supercritical fluid is a substance that, at temperatures and pressures higher than its critical point, shows compressibility, transportation, and penetration properties of a gas and the density and solvating power of a liquid. Additionally, in comparison to common solvents, supercritical fluids present higher diffusivities, lower viscosities, and almost null surface tensions, which provide them exceptional solvent and operational characteristics [123]. Finally, their properties can be easily tuned by changing the temperature, pressure, or even by adding a modifier (co-solvent). Supercritical carbon dioxide (SC-CO_2_) (Pc = 7.28 MPa, Tc = 304.1 K [123]) has been the most widely used fluid in SFE, since it is non-toxic, environmentally safe, non-flammable, low cost at high purity, and easily removed from final extracts. In addition, it allows the use of relatively low pressures and near room temperatures, which together with the absence of light and air in the process, reduces the possibility of oxidative degradation [124].

Several authors have applied SC-CO_2_ SFE for phenolic compounds from macroalgae (see Table 6). However, some studies have considered this technique less promising than others. Anaëlle et al., for example, observed a considerably lower TPC and antioxidant activity on SFE extracts when compared with those obtained by SLE or ASE [8]. Notwithstanding, this could mean that an optimization procedure should always precede the use of these novel methodologies. *Sargassum muticum*, as one of the most exploited macroalgae, has been also the object of several studies concerning the use of SFE [8,18,125]. Ethanol has been the most used modifier [8,125,126]. Conde et al. verified that the addition of 10% of ethanol (from a studied range of 0.5–10%) in the SC-CO_2_ SFE extraction of phenolic compounds from *Sargassum muticum* increased the TPC 1.5 times [125]. Anaëlle et al. used a similar ethanol content (12%) in the extraction of phenolic compounds from the same macroalgae species [8]. Short extraction times, namely 1 or 1.5 h, have been chosen by most authors [8,18,125,126], although considerably higher values were adopted for the extraction of phenolic compounds from red and green macroalgae [127].

Notwithstanding, kinetic studies performed in the SFE extraction of phenolic compounds from *Sargassum muticum* showed a maximum of both TPC and antioxidant activity at 40 min of extraction time [125]. The temperatures and pressures used in the SFE of phenolic compounds from macroalgae have ranged from 30 [126] to 60 °C [8] and from 8 [126] to 50 MPa [127], respectively (Table 6). Klejdus et al. observed a maximum recovery of several isoflavone standards using SC-CO_2_ SFE modified with methanol:water (10:90) at 40 °C (in a studied range between 35 and 75 °C) and at 35 MPa, which was then applied successfully to extract the same isoflavones from several macroalgae species [18]. The optimal conditions verified for the SC-CO_2_ SFE modified with 10% ethanol of phenolic compounds from *Sargassum muticum* were at 50 °C and 20 MPa [125], while the TPC of *Undaria pinnatifida* extracts obtained with SC-CO_2_ SFE modified with 3% ethanol were maximized at 60 °C and 25 MPa [126]. Nevertheless, it must be highlighted the few number of studies have already confirmed the presence of phenolic compounds in macroalgae extracts obtained by SFE [18,42].

It should be emphasized that one of the main weaknesses of the studies concerning the SFE of phenolic compounds from macroalgae is the lack of a detailed characterization of the polar fraction of the extracts obtained, being unknown the type of macroalgae phenolic components or families that could be extracted by this methodology. Some authors already reported the presence of phlorotannins in SC-CO_2_ SFE extracts, however a more detailed characterization of the lipophilic components has pointed out the high abundance of fatty acids in the extracts obtained (which might be related with the absence of a modifier) [42]. Therefore more studies have to be done in order to evaluate the efficiency of this methodology on the extraction of macroalgae phenolic compounds.

In addition to the exploitation of the above-mentioned novel methodologies, in the last years, the use of sustainable solvent media such as ILs and natural DES (NADES) by both conventional or by novel methodologies to extract valuable components from natural resources has emerged as a hot topic. ILs, salts with melting points below 100 °C, are usually composed of a large organic cation and an organic/inorganic anion, with a multitude of cation/anion combinations bestowing them with tunable properties (e.g., hydrophobicity, solution behavior). These “designer solvents” and particularly their aqueous solutions have shown a remarkable potential for the extraction of added value compounds [81]. Finally, NADES are emerging as remarkable solutions for bioactive compounds extraction [82] due to the diversity of combinations and their benign/sustainable nature. Nevertheless, ILs and NADES have not yet been explored in the extraction of phenolic compounds from macroalgae.

## 4. Analysis of Phenolic Rich Crude Extracts

Crude macroalgae polar extracts are complex mixtures, where not only phenolic components but also contaminant polysaccharides, proteins, and other polar metabolites co-exist in abundant diversity. While a detailed analysis of the structural diversity of the extract is hard at this point, an initial characterization by spectrophotometric assays, such as TPC by the Folin–Ciocalteu reaction, or TPhC by the 2,4-dimethoxybenzaldehyde (DMBA) assay, is frequently employed, mostly due to the simplicity, low cost, rapid execution, and potential for comparison with other works. However, the lack of specificity and the sensitivity to interferences leads to poor reproducibility/reliability, greatly discouraging the usage of these techniques as sole descriptors of the extractives’ identity; nonetheless, due to their spread use, an initial discussion is held on this topic in Section 4.1.

More reliable techniques for the analysis of crude extracts have been reported. Nuclear magnetic resonance (NMR), Fourier transform infrared spectroscopy (FT-IR), and ultraviolet–visible spectroscopy (UV–vis) have been extensively used, mostly as confirmative techniques, to improve the reliability on spectrophotometric measures—i.e., confirm the phenolic components’ presence in the extracts through more specific (yet rather uninformative when applied to crude extracts) spectral signals characteristic of these compounds, thus clarifying the extent to which TPC and antioxidant activity are biased by contaminants. A brief discussion on these spectral “clues” is held n Section 4.2. Interestingly, proton NMR (^1^H NMR) has been reported as a good method to quantify certain phenolic compounds in complex mixtures, and this application is also exposed in this review.

### 4.1. Global Analysis by Spectrophotometric Analysis

The spectrophotometric characterization of the phenolic rich extracts from macroalgae involves mostly the estimation of TPC and TPhC, although some authors have also estimated the content of other specific families, such as flavonoids or anthocyanins [66,67,92,102,128]. The most commonly used method to determine the TPC in macroalgae extracts is the well-known Folin–Ciocalteu method, firstly established as Folin–Denis method [129] and then modified by Folin and Ciocalteu [130] and later by Singleton and Rossi [131]. However, some authors have used other methods [132], namely, the Prussian Blue assay [133]. These assays are based on redox reactions, in which the phenolate ions are oxidized and the $F_{e}{\left(CN\right)}_{6}^{3-}$ anion (in the Prussian Blue assay), or phosphotungstic-phosphomolybdic (in the Folin–Ciocalteu assay) are reduced, forming colored products. However, in addition to phenolics, other free hydroxyl groups can participate in those reactions, and thus, these assays only give an estimation of the amount of phenolic compounds present in the extract. The lack of specificity of the Folin–Ciocalteu method has been described as its major limitation [134], with the possible contribution of non-phenolic compounds leading to an overestimation of the results. However, considering its use for comparative purposes, the main problem concerns in the lack of a standardized methodology. In fact, from the vast range of studies regarding the TPC of macroalgae extracts, there is a lack of consensus in what concerns the volume and concentration of extracts and reagents [28,55,65,128]. The incubation time has also been quite divergent, with some authors applying 20 [28] or 30 min [54,135] and others extending it up to 2 h [65,128].

Additionally, no consensus is found in the standard used for the calibration curves, which makes even more difficult the comparison between different studies. Most authors have expressed the TPC of macroalgae extracts as gallic acid [28,57,65,72,77,89,92,102,105,110,112,119,135] or phloroglucinol equivalents [104,136,137]. However, the use of other standards such as catechol [95,128] or tannic acid [54] has already been considered. As mentioned above, there is no specificity of the Folin–Ciocalteu method for phenolic compounds or even for phlorotannins. Therefore, some authors have inaccurately used the Folin–Ciocalteu method to estimate the TPhC (expressed as phloroglucinol equivalents) [60,79,80,87,108]. In fact, the comparison of the Folin–Ciocalteu and Prussian Blue methods to the DMBA assay, a colorimetric method described as specific for 1,3- and 1,3,5-substituted phenols, showed a great overestimation of the first two methods [64,132]. The reaction of DMBA and phlorotannins is based on an electrophilic attack by the aldehyde under acidic conditions, forming a pink colored chromophore. In fact, in the last years, several authors have accomplished TPhC estimation by this assay [9,58,70,96,104]. Consensus is found in the standard used, namely phloroglucinol, despite being suggested that TPhC is underestimated when this standard is used [132].

### 4.2. Direct Spectroscopic Analysis of Crude Extracts

Crude extracts are often too complex to be analyzed by high-resolution techniques. However, some spectroscopic techniques allow a rough estimation of both qualitative and quantitative composition through the presence of “diagnostic” signals. For instance, it is widely known that phenolic compounds absorb radiation in the ultraviolet (UV) region of the spectra (with absorption maximum between 260 and 330 nm), which is often used as an indicator for the presence of this family of compounds. However, the molecular absorptivity of derivatives of simple phenolic compounds is quite variable, and full spectra are highly influenced by media pH [138] or by the co-existence of other components.

Also, due to the high absorbance of proteins and nucleic acids in the same region, this technique is more relevant when working with standard compounds, or as a detector in chromatographic equipment, as discussed in the next section. A strategy for the confirmation of phenolic moieties UV absorbance has been to evaluate the existence of a bathochromic shift of absorption maxima towards longer wavelengths, as a result of phenolic groups ionization to phenolate upon the addition of concentrated NaOH solutions [139]. This method was used in the study of phenolic extracts from *Fucus spiralis*, where a shift of 16 nm in the absorption maximum of 270 nm peak was registered upon the addition of two drops of NaOH 2 M directly in the spectrophotometer cuvette [140]; this value was reproduced in the analysis of *Sargassum siliquastrum* extractives [141].

FT-IR has been a common method explored in the characterization of phenolic extracts. The presence of phenolic groups in macroalgae extracts has been assessed by the simultaneous occurrence of bands absorbing in the regions corresponding to hydroxyl groups (3000 to 3500 cm^−1^) and aromatic rings (1200 to 1700 cm^−1^ as well as 2850–3000 cm^−1^) [56,140,141,142,143]. Although not being yet reported in these studies, characteristic bands of C–O–C bonds of phlorotannins may also be observed in FT-IR spectra. Unfortunately, attention has to be paid to the possible interference of co-existing compounds, such as sulphated polysaccharides, namely fucoidan (S=O, ~1350 cm^−1^), widely present in brown macroalgae.

NMR allows a more detailed (albeit still very unspecific) analysis of complex mixtures, being widely used in the analysis if crude extracts or enriched fractions [7,8,25,34,61,64,91,144,145,146,147,148,149] Indeed, ^1^H NMR has been used to confirm the presence of phenolic constituents [8] as well as to detect [145] and quantify [147,149] phlorotannins. The protons from phenolic units have characteristic chemical shifts that can be used to detect the presence of phenolic moieties. Also, thanks to the resolution of ^1^H NMR, not only phenolic compounds but also carbohydrates can be detected, allowing a qualitative analysis of the extract’s purity. In an experimental setup for the optimization of green extraction processes for *Sargassum muticum* bioactive phenolic compounds, Anaëlle et al. managed to track the relative proportions of phenolic compounds (measured in the 5.5 to 6.5 ppm range) and of mannitol, a common co-extractive in phenolic extracts, by the signal obtained through the high resolution magic angle spinning (HR-MAS) resonance at 3.6–3.9 ppm (the region of polyols) [8]. Other studies with brown macroalgae have also used ^1^H NMR chemical shifts in the range of 5.5 to 6.5 ppm to confirm the presence of phenolic compounds and their relative quantity [145,148].

Parys et al. have used another NMR approach to quantify phenolic compounds [149]. More specifically, resonances in the region of 6.0–6.3 ppm (typical from phlorotannins’ aromatic protons) were integrated and quantified against an internal standard (trimesic acid). However, this study obtained highly over-estimated values for phenolic compounds concentration (at least compared to Folin–Ciocalteu calculated values, which are already assumed to be oversized). Jégou et al. have used ^1^H HR-MAS to detect phloroglucinol in *Cystoseira tamariscifolia*’s unprocessed biomass [147], and managed to develop a method for the quantification of this compound with 94.2% accuracy—unlike the previous study, one single resonance (that of the three C–H bonded protons in phloroglucinol) at 6.02 ppm, was used. Simultaneously, the Folin–Ciocalteu assay indicated values up to 30× higher, thus, much overestimated and thus in line with the discussed in the previous sub-section. The authors claimed that quantitative NMR can potentially be optimized to most compounds, becoming a practical, reliable method to assess the concentration of a metabolite in the complex mixture of a crude extract.

Carbon NMR (^13^C NMR) has also been used to assess the purity of phlorotannins-rich extracts [132,150]. In both studies, the assignment of given spectral profiles to phlorotannins was made by the comparison with published data from isolated compounds [47], on which common chemical shifts regions, namely, at 95–107, 123–134, and 143–164 ppm were identified.

## 5. Primary Fractionation of Macroalgae Polar Crude Extracts

In most cases, the study of phenolic compounds from macroalgae involves separation and purification steps, for the fractionation and/or isolation of the compounds of interest, and the subsequent analysis for structural characterization by spectroscopic techniques. In fact, those studies involving direct structural characterization of crude extracts are unquestionably compromising the number of components detected/identified [57]. Primary fractionation of the extracts precedes most analytical studies, resulting in the separation of the extracts into fractions according to molecular weight, charge, chemical affinities, or solubility. Such processing is intended to reduce complexity, allowing further high-resolution chromatographic separation steps, but also to prevent damage to the columns and remaining components of the instruments. Thus, studies of both approaches were included in this review. From 85 of a total of 99 peer-reviewed articles analyzed, data on solubility-based separations (liquid–liquid extraction and solutes precipitation), adsorption-based separation (including solid-phase extraction, SPE), particle-size-based separation (molecular-weight cut-off dialysis, MWCOD; and ultrafiltration, UF) and molecular-charge-based separation (capillary electrophoresis, CE) were gathered, and are discussed below.

### 5.1. Solubility-Base Separation

Liquid–liquid extraction (LLE) is by far the most common, and firstly applied, method to phenolic rich crude extracts. The extraction of macroalgae phenolic compounds is often conservative, i.e., along with the phenolic components, other molecules are co-extracted, such as polysaccharides, proteins, and some medium-polarity lipophilics (e.g., carotenoids). For that reason, the partition of these distinct classes in carefully chosen solvents obtains polarity-segregated fractions. LLE has been essentially reported for the isolation of phlorotannins, which, besides resulting from the higher amount of studies with phlorotannins comparing with those of miscellaneous phenolic compounds, is also a result of the broad range of polarities that miscellaneous phenolic compounds present.

In fact, a lot of experimental data exists on the fractionation of macroalgae phlorotannins [40,60,61,76,143,151,152,153,154]. Some studies have reported the fractionation through a single LLE step. Tierney et al. simply washed ethanolic brown macroalgae extracts with water, which was frequently replaced (top layer decanted), obtaining phlorotannin rich fractions which were further submitted to other fractionation and isolation methodologies before analysis [78]. Similar approaches were used by other authors [28,155]. For instance, *Cystoseira abies-marina* phlorotannins enriched extract was successfully obtained by simply defatting the crude extract with dichloromethane [36]. In other studies, the partitioning of the phlorotannins-enriched fraction in a single ethyl acetate LLE has been equally sufficient for further fractionation with adsorption-based techniques [156,157]. However, the majority of LLE approaches have used sequential fractionation, initiated by removing the hydrophobic components extracted, using hexane, chloroform, dichloromethane, light petroleum, or a combination of these [60]. The remaining hydrophilic fraction (from the solvent of extraction) was usually treated with ethyl acetate (to which phlorotannins get partitioned), and in some cases further washed with butanol [101]. Some authors have used methanol [69] or ethanol [119] to precipitate carbohydrates, while Glombitza et al. used light petroleum to precipitate the high-molecular-weight fraction of an acetylated phlorotannins extract previously partitioned into ethyl acetate [40]. Other authors have combined different LLE steps in order to characterize phenolic compounds without any further fractionation. Catarino et al. submitted a crude acetone:water extract of *Fucus vesiculosus* to a first LLE with n-hexane to remove hydrophobic components followed by partitioning with ethyl acetate, proceeding to the characterization of phlorotannins by mass spectrometry (MS) coupled to ultra-high-performance liquid chromatography (UHPLC) [9]. Finally, the fractionation with ethyl ether followed by ethyl acetate of brown macroalgae ethanol:water extracts was enough to detect and identify by HPLC-MS a high number of miscellaneous phenolic compounds, including phenolic acids, flavonoids, and their glycosides and phlorotannins [19]. It must be highlighted that few authors have reported using LLE to obtain miscellaneous phenolic compound enriched fractions. In fact, only one additional study has used LLE in the processing of an extract for whole-phenolic compound analysis [17]. The crude methanol 60% (aqueous) extract of *Himanthalia elongata* was partitioned using ethyl acetate, a medium polarity solvent often used for phenolic compounds. Effectively, meta/para-hydroxybenzaldehyde, phloroglucinol, gallic acid, kaempferol, cirsimaritin, gallic acid 4-*O*-glucoside, and carnosic acid were detected in the ethyl acetate fraction. A note on the recovery of miscellaneous phenolic compounds is now required: first, one must account that the fact that phenolic compounds partition into the organic extract (to some extent), while other contaminants do not (such as polysaccharides), is what makes relatively apolar solvents suitable for this kind of purification at the expense of losing a significant amount of phenolic extractives in the aqueous residues; second, one must consider the fact that biological matrices are much more concentrated in hydrophilic substances (sugars and proteins) than in phenolic secondary metabolites, and for that reason, crude alcoholic/aqueous extracts, even though ideal for phenolic compounds solvation, will readily lose these components when fresh solvent (one that can solvate the phenolic compounds) is added in large volumes (far from saturation). Once again, external factors affect the behavior of phenolic substances in solution, with an impact in their laboratory processing: the concentration of more hydrophilic contaminants, and the compromise that the researcher needs to establish between quantitative or qualitative recovery.

### 5.2. Adsorption-Base Separation

Adsorption based separations are an emerging technology due to their simplicity and the potential for scale-up and higher specificity compared to other primary fractionation techniques [158]. Separation of compounds is achieved by mixing the solubilized extract with a solid matrix, to which the target compounds and the unwanted contaminants have distinct affinities. Thus, specific compounds can be recovered by separating the solid and liquid phases (e.g., by reduced pressure filtration), and processing the one known to contain the desired compounds (i.e., further processing the supernatant, if the target metabolites are not adsorbed, or eluting the analytes from the matrix, if they do adsorb).

In the case of macroalgae phenolic compounds, three matrices have been reported to adsorb particularly phlorotannins. Diaion^®^ HP-20 is a hydrophobic, synthetic resin, widely used to adsorb compounds with hydrophobic moieties. Columns filled with this matrix were already used to enrich ethanol:water (70:30) water [159] and methanol:water (80:20) [116] extracts in phlorotannins. In this case, salts and hydrophilic compounds (such as sugars and proteins) are eluted with water, while phlorotannins get adsorbed and released only after rinsing with ethanol:water or methanol:water mixtures. Another relatively nonpolar matrix, namely polyvinilpyrrolidone (PVP), has been used to purify sulphated phlorotannins of *Pleurophycus gardneri* [39]. In this work, a suspension of PVP particles was mixed with an acidified phlorotannins aqueous fraction (pH 5.5); the acidic pH allowed the adsorption of the sulphated molecules, while keeping them protonated and thus less polar. Consequently, after washing with water, the recovery of the sulphated phlorotannins was achieved by elution with aqueous NH_3_ (pH from 8 to 11) due to the deprotonation of the acidic sulphate moieties.

Several works have also proposed cellulose as an adsorbent [35,70,132,160,161,162]. This material has revealed high affinity for phlorotannins, allowing the washing out of residual lipophilics and salts without a significant loss of the target analytes. One approach to this adsorption setup is to evaporate a methanolic solution of the crude extract after stirring with cellulose (often, microcrystalline cellulose “Avicel” is used), and further rinse it with toluene, discarding pigments, and, with aqueous acetone (66–70%), recovering the phlorotannins [35,70,132,160]. Another approach is the lipophilic removal prior to the adsorption step [161,162], using cellulose as a media to wash off salts and other contaminants, which can be done by solubilizing the sample in methanol 50%, thus solvating the contaminants as well, and treating the extract with cellulose, which is then removed by filtration or centrifugation. The remaining liquid can be discarded, and phlorotannins recovered by re-washing the cellulosic material with methanol:acetone 2:1.

Cellulose can be a very interesting matrix for further research, as it has the potential to be industrially used, in a sustainable valorization of industrial by-products, without compromising the applicability of the extracts for the food industry, given its biocompatibility. Although recovery of more than 75% of the weight of the crude extract was reported [162], suggesting a reduced loss of analyte, optimizations of adsorption methods are still required to increase specificity and reproducibility.

Solid phase extraction (SPE) is another adsorption-based separation technique, in which the adsorbent is immobilized in a cartridge, allowing the sequential elution of compounds to occur with gradient solvents. The size and nature of SPE cartridges make it more suitable to binary separations, i.e., it is typically used to receive an extract and split it into two polarity- and/or charge-opposed fractions. Similar to chromatography, SPE can be used in either normal or reversed phase. In addition, several commercial products exist, and most are adapted for the acceleration of elution with vacuum/reduced pressure systems.

Phenolic compounds have been processed by RP-SPE several times [10,58,63,77,88,144,163,164]. Although C18 solid phases have always been used, the application of different elution schema allows different processes to be drawn: distribution of phlorotannins between the washing fractions and the posterior eluted ones will depend on the initial solvent of resuspension, since the phase of application of the sample to the cartridge will define what molecules get eliminated in the run-through and to what extent the phlorotannins will adsorb in the octadecyl matrix. For instance, the RP-SPE of phlorotannins extracts can eliminate the hydrophilic contaminants by resuspension of the extract and washing of the column with water, eluting minerals, proteins, and sugars in these fractions, while phlorotannins stay retained due to the hydrophobic interactions with C18 [10,144]; posteriorly, their recovery can be made by eluting the cartridge with 50% [144] or 100% [10,58] methanol, which is nonpolar enough to desorb these compounds. Some authors actually have improved the process by acidifying the solvents (both the water in the resuspension/washing phase, and the organic solvent, methanol [63] or acetonitrile:water (80:20) mixture [88]), protonating, respectively, the phenolic acids and flavonoids and phlorotannins, which despite improving their solubility in water, also improves their retention in hydrophobic media by reducing the polarity in acidic hydroxyl groups. Simple phenols and phenolic acids of *Padina gymnospora* have also been purified by a Sep-Pak C-18 cartridge (Water Associates, Millipore™, Billerica, MA, USA) after resuspension in water, thorough washing, and elution of adsorbed phenolic compounds with ethanol:water (30:70) [164]. Also, a method for the extraction and purification of these classes of compounds was suggested by Onofrejová et al. consisting of a pressurized liquid extraction followed by an SPE. In this study, the authors demonstrated that a special mixed mode (reverse-phase/ion-exchange) SPE cartridge (Oasis MCX, 60 mg) allows the concentration and purification of a diverse set of phenolic components by means of pH manipulation of the eluent. More noticeably, the method allowed the downstream analysis of the fractions by HPLC-MS in the sub-nanogram scale [72].

The application of normal-phase SPE has only been reported once for macroalgae phenolic compounds [165]. After several purification steps (including LLE and column chromatography) without sufficient removal of polar contaminants for phlorotannins ESI-MS analysis, *Ascophyllum nodosum* extract was ressuspended in methanol:chloroform and passed through a silica-filled cartridge [165]. Elution was carried out first by passing the same solvent, and afterwards by washing with methanol. Phlorotannins with enough purity to obtain good signal peaks in ESI-MS were obtained in the first fraction (methanol:chloroform), probably because the polar contaminants had little solubility in this solvent mixture, and became adsorbed to the silica, while the hydrophobicity of phlorotannins was sufficient to maintain them solvated and repelled from such a polar matrix. Even though NP-SPE was only applied once, the abundant reports on normal-phase chromatography suggest this phase can be successfully employed in a cartridge-scale, adsorption-based fractionation of macroalgae crude extracts.

### 5.3. Other Primary Separation Methods

Other separations of complex extracts have been performed prior to preparative or analytical chromatography by means of ultrafiltration (UF) [80,120,140], molecular-weight cut-off dialysis (MWCOD) [28,78,166], a combination of these two [148], and capillary electrophoresis (CE) [167]. UF and MWCOD are techniques that segregate compounds by their molecular weight (MW) with less instrumental requirements, time, expertise, or investment than the alternatives. In fact, with UF/MWCOD, a clear-cut separation of the molecules around a defined limit of MW can be achieved, allowing easy separation of discrete fractions over a wide range of MWs with a few combinations of membranes/filters.

Isolation of the low-molecular weight phlorotannins (LMWP) from *Fucus vesiculosus*, *Fucus serratus*, *Himanthalia elongata*, and *Cystoseira nodicaulis* was carried out using membrane dialysis with a MWCO of 3.5 kDa (meaning a maximum DP—slightly above 25) [28]. The study of phlorotannin profiles in macroalgae has often been reported in terms of the number of PGU. In fact, as DP gets higher, the number of isomers grows drastically, preventing common MS approaches of distinguishing them. Phlorotannins with a DP between 2 and 10 are often reported as LMWP; however, by separating the phlorotannins pool at the 3.5 kDa cut-off, phlorotannins of 16 PGU could be analyzed by the chromatographic platform [28]. Nonetheless, most antioxidant activity remained in the >3.5 kDa fraction. This is because although LMWP are much more described in literature, the high-molecular-weight phlorotannins (HMW) are expected to be much more abundant in macroalgae biomass. The same rationale—of separating ranges of molecular weights for a more efficient analysis—was used by other authors, but the >3.5 kDa fraction was further dialyzed with a 100 kDa cut-off membrane, producing, therefore, the fractions of 3.5–100 and >100 kDa [78]. Spectrophotometric measures suggested that the 3.5–100 kDa was the richest in phenolic compounds, which is in agreement with the expected distribution of phlorotannins molecular weight. The fraction higher than 100 kDa is thought to retain most polysaccharides, contributing to such enrichment; on the other hand, while LMWP are dialyzed into the <3.5 kDa fraction, so is one of the major contaminants in macroalgae phenolic extractives—mannitol—which leads to the lower values of TPC and antioxidant activity of this fraction. Nonetheless, by applying further purification (namely, reversed-phase chromatography), the authors managed to obtain fractions containing phlorotannins up to 25 PGU, as analyzed by MS. This study reinforced the notion that besides the <3.5 kDa fraction containing many different phlorotannins to be analyzed, the non-analyzable fraction of 3.5–100 kDa actually corresponds to a rich, biologically potential extract.

Similarly, UF has been used to generate MW-dependent fractions, alone or in combination with MWCOD. The separation of *Fucus spiralis* phenolic extract into three fractions (F1 < 1 kDa < F2 < 3 kDa < F3) using a series of filters also demonstrated that functional assay results (namely ACE-inhibitor activity) are improved in the fraction of >3 kDa [140]. The lower MW fractions probably contained phenolic compounds as well (as suggested by their FT-IR spectra), but the higher concentration of small organic and inorganic contaminants might explain their lower activities. Other reports of similar studies, illustrating the inclusion of UF in the fractionation of phlorotannins [80,148] or other phenolic compounds [120], can be found.

CE has been successfully used to separate catechin and gallic acid from a *Fucus vesiculosus* extract [167]. The profile was obtained in less than 1100 s. This method is an alternative to chromatographic setups and is highly appreciated for its rapidity, small scale, and low solvent waste. It has also been suggested as a good method to analyze polar extractives of plant and macroalgae matrices, because of its capacity to separate sugars and phenols [168]. Despite not being a preparative purification method, the very small usage of samples and the very short time to obtain results make CE an excellent candidate for routine analysis of extracts along the process of purification to monitor the qualitative and quantitative changes in between steps.

## 6. Preparative Chromatographic Techniques for Phenolic Compounds Isolation

Although some studies have already structurally characterized macroalgae phenolic compounds without time-consuming purification methodologies, their use has been particularly useful whether for in depth MS and NMR analysis or even for biological activities evaluation. In fact, macroalgae crude extracts obtained with water, alcohols, or mixtures of both are often highly concentrated in polysaccharides, as well as in proteins. Even other less polar contaminants such as carotenoids are often found to partition into alcoholic solvents easily. Therefore, phenolic components need to be isolated prior to most analytical approaches. While more modern techniques—based on chromatographic-mass-spectrometric platforms—allow the simultaneous separation and identification of organic compounds, such analysis can only become routine once enough data on structural diversity and mass spectra have been gathered for individual compounds.

The characterization of phenolic constituents by spectroscopic analysis of pure compounds is highly dependent on the success of preparative chromatographic separations, from which isolated compounds can be recovered. The methods for this fractionation are analyzed in this section.

### 6.1. Column Chromatography

In spite of the reduced capacity for handling complex samples in low quantities, limiting its applicability in fractionation for structural characterizations, preparative column chromatography (CC, also noted as LC by some authors) is still a very useful technique to remove impurities. In fact, these undesired components can make posterior analysis difficult or even narrow the range of compounds to a sharper variation of a given physical–chemical trait (polarity, molecular weight, etc.), as well as cause possible damage to the HPLC equipment. In macroalgae phenolic compounds analytical processing pipelines, CC has been used in normal phase (more often), reverse phase, and size-exclusion separation modes. Table 7 lists the CC application to macroalgae extracts.

Normal phase CC (NP-CC) has been amongst the most used stationary phase, particularly to isolate macroalgae phenolic compounds (see Table 7). Effectively, silica gel fillings for column packing are one the most widespread and accessible materials, justifying the routine application of often multiple NP-CC to the same extract, allowing successive adjustments of eluent and, therefore, of separation power. Silica gel is thought to interact with phlorotannins and other phenolic components by hydrogen bonding between hydroxyl groups of both molecular structures [34]. Thus, it is logical that more hydroxylated compounds get retained longer, and that increasing polarity of the eluent results in the sequential elution of compounds. Depending on the range of polarities of the metabolites on the extract, more or less accentuated gradients of polarity in the eluent can be used—an overview of the mobile phases used in NP-CC is displayed in Table 7. This type of stationary phase has been often employed to separate the ethyl acetate fraction after LLE [32,76,157,169,174,182], since this fraction might still contain metabolites soluble in ethyl acetate, that is likely to be more rapidly eluted than phlorotannins, which, despite the bulky structure, present many hydroxyl groups on their surface. Thus, with a very simple and inexpensive combination of methodologies, some extracts might be practically narrowed to the phenolic fraction. In a study concerning the phlorotannins analysis from *Laminaria digitata*, NP-flash CC was successfully used to provide fractions for UHPLC-MS and matrix-assisted laser desorption/ionization time-of-flight mass spectrometry (MALDI-TOF-MS) characterization [44]. Conversely, in other cases, NP-CC-originated fractions were further fractionated by RP-CC [32,76,172], SEC-CC [157], or thin-layer chromatography (TLC) [56], providing bi-dimensional resolution of the components from the initial extracts and resulting in isolated compounds. The NP-CC of a methanol:water extract of *Dasycladus vermicularis*, followed by SEC-CC also resulted in two isolated coumarins [21]. Actually, in some cases authors used several sequential steps to provide a high number of isolated phenolic compounds, such as the study concerning the phlorotannins profile of *Ecklonia cava*, in which a UAE methanol:water extract (80:20) was submitted to different RP-CC, SEC-CC, and preparative HPLC, allowing the isolation of 8 phlorotannins [116].

Reversed-phase CC (RP-CC) is not as common as NP-CC. While silica gel is widely available, RP stationary phases use more specific products that are not so ubiquitous. Besides, RP bench applications have been popularized as SPE cartridges, and high-resolution RP is nowadays a common configuration of HPLC equipment. Thus, the use of either RP-SPE or RP-HPLC combined with NP-CC provides two dimensions of separation that, in many cases, replace the need for column approaches. Nonetheless, some studies have reported the use of RP-CC of phlorotannins [32,76,151,159,172,177,178,182] or coumarins and phenolic acids [21], mostly with octadecyl-derivatized stationary phases. This matrix is the most widespread RP phase since it provides excellent resolution for a wide variety of organic compounds while using aqueous and low toxicity polar solvents for elution (instead of the hydrophobic ones associated with NP-CC, which are costly, toxic, and polluting). Effectively, aqueous methanol has been the only mobile phase used for phenolic compounds RP-CC (see Table 7), using gradients of decreasing polarity (by increasing methanol percentage in water).

At last, size exclusion chromatography (SEC-CC) using Sephadex LH-20 has been repeatedly used in the fractionation of macroalgae polar extracts [60,76,77,88,115,151,156,157,174,175,177,178,179,180,181,182] The separation of compounds in Sephadex LH-20 is promoted by the metabolites’ molecular size, but also by its solubility in the eluent. This gel-filtration media is a reticulated network of dextran derivatized with hydroxypropyl groups, such that both hydrophilic and lipophilic regions exist in the surface of the mesh. Along elution, an extra dimension of separation is introduced by the interactions of the analytes with the media, so typical gradient elution allows an augmented separation. Effectively, SEC-CC has been used both at the beginning of purification procedures, to separate polyphenolic compounds from simpler, low molecular weight polar metabolites, and in the final steps of fractionation, rendering HPLC-suitable fractions or even isolated compounds.

### 6.2. High-Performance Liquid Chromatography

HPLC is a chromatographic technique in which the stationary phase is composed of solid particles of such reduced dimensions that surface area and consequently retention time are greatly improved; silica, coated silica, or polymers are examples of materials packed into an HPLC column, and the mobile phase can be any suitable liquid [183]. The increased compaction of the column creates a need to pressurize solvent pumping, which led to the high-pressure alternative designation of the initial HPLC. The successful use of HPLC involves the choice of an appropriate column, mobile phases, and a detector [183]. Apart from the different columns and mobile phases, a photodiode array (PDA) or a simple UV detector have been widely used to detect and, in few cases, to quantify phenolic compounds from macroalgae upon chromatographic separation (Table 8).

Normal-phase HPLC (NP-HPLC) was more commonly used for phlorotannin isolation in the past. In fact, many of the studies of Glombitza and colleagues (Table 8), where a vast amount of phlorotannins were firstly isolated and described, achieved their purification using NP-HPLC [40,41,43,45,46,154,171,184,185]. Adding to these results, there are strong theoretical considerations supporting the use of NPs in phlorotannin separation, suggesting this type of column might be equally useful to isolate these molecules as the more modern C18-based RPs. The use of proper elution conditions, and the previous acetylation of the phlorotannins, might be key factors for their separation. Interestingly, the various reports of NP-HPLC describe a phenomenon of elution according to DP, suggesting that bigger molecules, for presenting more hydroxyl groups on their surface (or acetyloxy-groups, which also provide nuclei for hydrogen bonding), become retained longer in the silica-based columns [34]. In fact, elution with increasing concentrations of ethanol in chloroform, albeit to low ethanol final percentages (2–3% *v/v*), was the primary choice of eluent regardless of the phlorotannin type. This is because interaction with silica, and sequential disruption of this interaction by ethanol, is not dependent on (or sufficiently sensitive to) the variations in polarity among a given DP. The NP-HPLC separation of a fuhalol-enriched fraction from the macroalga *Sargassum spinuligerum* allowed the elution from the smallest to the largest DP [154]. In this paper, the authors also point to the fact that the more abundant phlorotannins are consistently of an even number of PGUs. Because the chloroform–ethanol system has been used, a given DP is represented by a relatively well-defined peak, although different isomers were co-eluted. 

Separation of a given DP fraction in the same column was done by changing the eluent (re-injecting the collected fraction of the chloroform:ethanol system and eluting it with chloroform:hexane, decreasing the concentration of the latter) [40,46]. This way, a more “fine-tunable”, slow separation of the compounds exists, and the overall low polarity of the eluent during the entire chromatographic separation ensures the solvent is not disruptive for the silica-phlorotannin interactions, increasing resolution of isomers. This approach allowed the separation of brominated phlorotannins of *Cystophora congesta* [46] and trihydroxyphlorethols of *Carpophyllum angustifollium* [40]. Noteworthy, this resolution is particularly amazing since NP-HPLC of halogenated phlorotannins has been shown to be difficult due to very high retention, while in this case, isomers, where only the position of the halogen differed, are resolved [46].

Reversed-phase (RP) HPLC columns have been the most used in macroalgae phenolic rich extracts [32,39,62,73,157,159,169,173,180,182,186,187,188,189]. Octadecyl (“C18” or “ODS”) has been the most used stationary phase, with a particle size of 5 µm. Several commercially available C18 columns, with varying internal diameters and lengths, have been shown to separate phlorotannins (see Table 8). Elution of these molecules from reversed phases has been performed using either water, methanol, or acetonitrile in different combinations. Although water to acetonitrile has been the most frequently used eluent, water to methanol gradients have been also used with success [21,157,187,188], which is an advantage since methanol is relatively easy to evaporate, and is much cheaper than acetonitrile. Nonetheless, acetonitrile might provide greater resolution at lower percentages, and might be adequate to use in columns of lower maximum pressures [183].

More interestingly, although elution with non-pH-adjusted solvents has been successfully achieved, the addition of trifluoroacetic acid [73,189], formic acid [32,116,159,169,186], or acetic acid [62,91] has been reported in accordance to general RP-HPLC standard practices.

No study was found to compare the performance of RP-HPLC with and without acid for the same conditions. When acid is not used for the sole purpose of column maintenance, its effect on retention times should be addressed and optimized.

Finally, size exclusion chromatography (SEC) columns suitable for HPLC conditions have been used in the preparative separation of different molecular weight fractions from *Ecklonia stolonifera* extract [32,169], as well as to confirm the molecular size of an isolated metabolite from *Padina gymnospora* using two standards [164]. Isocratic elution was performed in both cases, using either 100% methanol or 100 mM sodium acetate buffer (pH 6.3, 20 mM EDTA, 250 mM NaCl), since fractionation was to occur only by molecular size, and no chemical interactions were to be differentially explored.

### 6.3. Other Preparative Chromatographic Techniques

Besides the more common column approaches to liquid chromatography, other chromatographies have been used, with different specificities, in the analysis of phenolic components from macroalgae. For instance, thin-layer chromatography (TLC) was widely used as a complementary technique to other chromatographies [56,61,116,190], either as a method to track the evolution of the extracts composition or as a preparative separation of a given class of compounds by scraping the silica after elution and recovering the compounds by CC. TLC is a very common method in organic chemistry, and even in the articles that reported using it, no detailed description of the conditions was performed—only the ubiquitous use of silica gel plates. The revelation of the chromatograms and elution are standard procedures that can be easily found in the literature (see [191,192]) and are, therefore, not covered in this review.

Solid support free liquid–liquid chromatographic techniques have been already used to purify phenolic compounds from macroalgae crude extracts or fractions. One study accomplished the separation of phlorotannins by centrifugal-partition chromatography (CPC) [33]. This type of chromatography uses liquid stationary and mobile phases; the latter maintained in the “column”—a rotor—by centrifugal force from rotation. As the mobile phase—the sample, immiscible with the stationary phase—is injected through an online series of rotors, each passage of the mobile phase will carry the compounds with less affinity for the stationary phase and sequentially lose those with higher affinities in the first rotors. By using a two-phase system of *n*-hexane:ethyl acetate:methanol:water (2:7:3:7, *v/v*), bioactive phlorotannins of *Ecklonia cava* have been isolated (purity > 90%), which represents an improvement in the operational ease with which purified fractions of the phenolic extracts of macroalgae can be obtained [33]. Zhou et al. were also able to isolate the phlorotannin eckmaxol from an *Ecklonia maxima* ethanol:water (80:20) extract using high-speed counter-current chromatography (HSCCC) after previous Sephadex LH-20 gel fractionation with dichloromethane:methanol (1:1) [115]. A two-phase solvent system comprising n-hexane/ethyl acetate/methanol/water (2:8:3:7, *v/v/v/v*) was used.

## 7. Phenolic Compounds Identification

### 7.1. High-Performance Liquid Chromatography-Tandem-Mass Spectrometry

HPLC and its higher-performance variants (UHPLC) are the most powerful tools for analytical chemists and biochemists to separate complex mixtures of biomolecules and/or secondary metabolites rapidly, efficiently, and with a low amount of sample. Analytical U/HPLC is very similar to those previously reported for fractionation, especially regarding the stationary and mobile phases. The major operational difference that can be found in the column in terms of dimensions, i.e., length, diameter, and internal particle size. In the case of analytical-scale HPLC, the low amount of sample needed to reach the detector allows very small volumes of extract to be analyzed, allowing studies of chemical composition even when biomass availability is limited. Liquid chromatography is often coupled to UV–vis detectors—mostly PDA—as this type of detection is instrumentally simple, widely applicable to a large set of molecules, and can be used for quantification (from Beer–Lambert’s law, given linearity). However, UV–vis detectors fail to provide unambiguous identification for each peak, as the different extractives being separated are often similar, and their response to radiation in this range of wavelengths indistinguishable. Alternatively, the use of mass spectrometers directly coupled to U/HPLC instruments have revolutionized the capacity to analyze complex extracts in a relatively short period of time. Nonetheless, it is important to note that UV–vis detectors are often present, even if MS is present as well since it helps in the interpretation of the chromatograms and the real-time tracking of the elution.

U/HPLC-coupled MS instruments are commonly used with electrospray ionization (ESI). This is understandable since this ionization method is adapted to receive a liquid stream of the sample and continuously deliver it ionized onto the analyzer. Interesting considerations can be drawn by analyzing the U/HPLC-MS configurations used for macroalgae phenolic compounds characterization (summarized in Table 9). Although the increasing trend on the attention for macroalgae phlorotannins, some other phenolic components have been successfully separated and identified using HPLC: the 60% methanol extract of *Himanthalia elongata*, for instance, has been screened for antioxidant activity and the most active sub-fraction after CC eluted with ethyl acetate was subjected to RP-HPLC [17]. A triple quadrupole (QqQ) was used to perform precursor ion fragmentation (usually, the pseudomolecular ion, [M-H]^−^), and neutral losses scan allowed the identification of 8 phenolic components of distinct classes (hydroxybenzoic acid derivatives, flavonols, flavones, phenolic terpenes, and others). A similar procedure was used to separate and identify 41 miscellaneous phenolic compounds from different brown macroalgae [118]. Apigenin and gallic acid have also been separated and identified from red macroalgae extracts (*Gracillaria birdiae* and *Gracilaria cornea*) by a linear methanol-to-aqueous formic acid 0.1% (*w/v*) gradient in a C18 stationary phase [193].

Onofrejová et al. developed and validated an RP-HPLC coupled to a single quadrupole (Q) method to separate phenolic acids and aldehydes from red and brown macroalgae polar enriched extracts [72]. Using the spectrometer at single ion monitoring mode (SIM), it was possible to obtain the limit of detection (LOD) and limit of quantification (LOQ) values at sub-nanogram scales. NP-HPLC of several macroalgae species was carried out using a Zorbax SB-CN column (amide-derivatized stationary phase) [18]. By applying fragmentation voltages selected for individual compounds, along with Multiple Reaction Monitoring (MRM) data acquisition mode, 8 isoflavones (daidzin, genistin, ononin, daidzein, sissotrin, genistein, formononetin, and biochanin A) were detected for the first time in macroalgae, even at the fentogram per injection scale.

**Figure 5:**
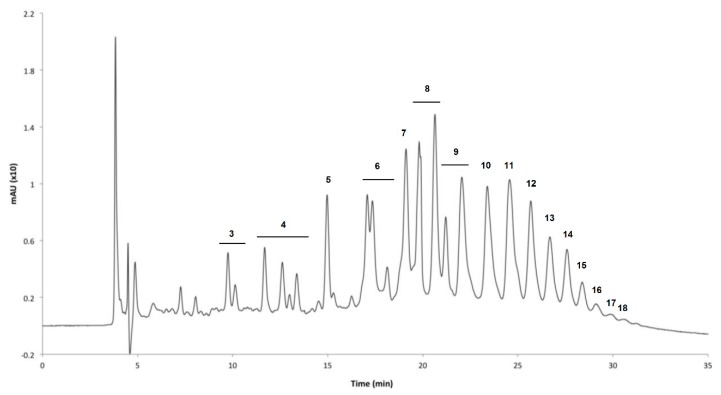
UV–chromatogram of *Fucus distichus* phlorotannins separated by degree of polimerization (numbers above the peaks) in a Develosil Diol column, using acetonitrile and 97:3 methanol:water. Detection at 254 nm. Adapted from [197].

Actually, RP-HPLC was the most frequently employed method for macroalgae phenolic compounds separation prior to electrospray ionization (see Table 9). The reversed-phases became popular for increased resolution of many organic compounds, but also for the shift in mobile phase composition, from non-polar to polar solvents—often water or aqueous mixtures [194]. The most common RP stationary phase is C18/ODS, which is composed of packed silica-bonded octadecyl chains. These chains provide a hydrophobic moiety that retains the more hydrophobic compounds. Most RP-U/HPLC applications in Table 9 have been performed under this type of stationary phase, as well as most non-MS-coupled preparative HPLC of phlorotannins. In a recent study, 84 different phenolic compounds, including phenolic acids, flavonoids, and phlorotannins up to octamers, from three different macroalgae species were detected and separated for further MS analysis using the same C18 column and the same gradient elution, with 1% aqueous formic acid and acetonitrile [19]. Vissers et al. successfully detected and separated phlorotannins from *Laminaria digitata*, after the NP-flash CC, with DP between 2 and 18 using an ethylene bridged hybrid (BEH, Waters) C18 column [44]. This type of column also allowed the separation of 4 and 6 phlorotannins from Ecklonia cava [152] and Sargassum palladium [195] extracts, respectively. Another common matrix used in RP chromatography of phlorotannins is pentafluorophenyl (PFP). This phase has been hypothesized to provide a basis for efficient separation of phlorotannins since its mechanism of retention is highly propense to interaction with these polyphenols: five atoms of fluor attached to a phenyl ring originate an electron-deprived aromatic moiety which is likely to interact with the phenolic moieties, and differentially retain structural isomers [36]. In fact, Macherey-Nagel GmbH & Co. KG has publicized their PFP bonded phases as an alternative to C18 for the separation of phenolic isomers—achieving perfect resolution of meta- and orto-cresol, and of the various isomers of dimethylated—or dihalogenated-phenols [196]. In fact, the studies that used PFP-bonded phases achieved consistently good resolution of phlorotannin peaks [28,36,165,166]. Montero et al. [36] actually compared C18 and PFP separation under the same experimental conditions. While the profiles obtained were both of good resolution, and qualitative differences were obtained—showing the techniques to be complementary rather than alternative—C18 still performed better than PFP (see Figure 5).

Despite the vast majority of studies employing RP, some authors claim NP could perform separation of phlorotannins because the abundant hydroxyl groups could provide a basis for differential elution [18,34]. Once a chromatographic setup has been optimized, NP and RP should, in theory, perform similarly (with inversed elution order of compounds); however, the poor diversity of stationary phases and other operational conditions for NP-HPLC—for instance, the need for less polar, organic solvents—make it unattractive and inadequate for ESI ionization. Also, past experience with NP separations of phlorotannins has shown that medium and high DP phlorotannins could not be resolved properly. For that reason, even though NP-HPLC could, in theory, be used, it was only reported once in the MS analysis of phlorotannins, and effectively, the LMW fraction of *Fucus vesiculosus* phlorotannins was well resolved [34].

Hidrophilic interaction (HILIC) stationary phases have been used, solely or prior to C18 separation in a two-dimensional apparatus [10,36,104,194]. Similar to NP, HILIC columns interact with compounds of high polarity, but the elution systems used are of mixed polarity, such that gradients lead to an internal partition of the solvent in the column, and of the compounds in the solvent phases [10]. Nonetheless, HILIC, as well as NP, are ineffective to separate high-MW components, probably due to their high retention [10]. Yet, HILIC-UHPLC (using an amide-functionalized stationary column) has managed to separate phlorotannins with DP up to 49 [10]. Also, HILIC-RP-2D-HPLC has been developed to characterize phlorotannins from *Cystoseira abies-marina* [36,194] and *Sargassum muticum* [104]. The resolution in the 2D separation was largely improved compared to HILIC or RP alone (up to 73 compounds separated in one run [104]). Negative mode ESI followed by an ion trap allowed the identification of the majority, and in the case of *Sargassum muticum*, the subclass of phlorotannins was tentatively assigned [104]. With an upgrade in the mass spectrometric determination of phlorotannins, 2D-HPLC configurations might be very useful to distinguish isomers (several resolved peaks with the same DP were found in both species [36,104,194]). Finally, size-exclusion HPLC (SEC-HPLC) was carried out with a Develosil Diol 250 mm column on brown macroalgae extracts, followed by IT-TOF hybrid MS, allowing the separation and identification by DP of several phlorotannins (Figure 5) [197].

#### Phlorotannins Mass Spectrometry

**Figure 6:**
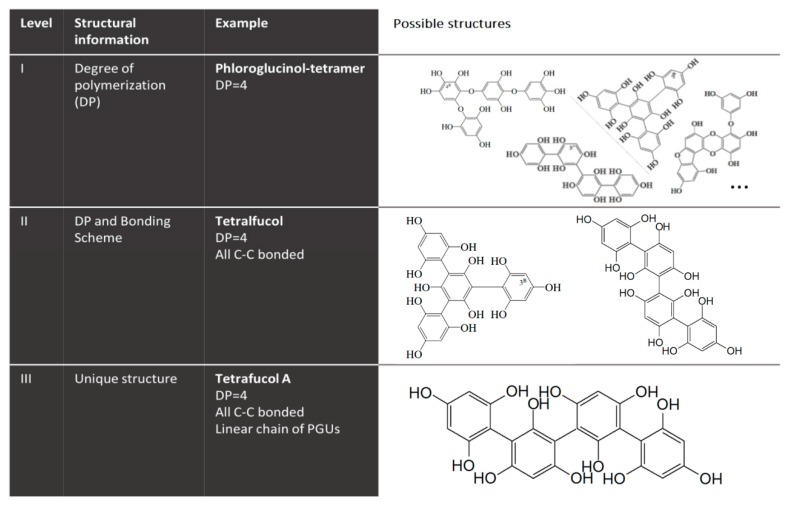
Schematic representation of the three levels of structural detail in phlorotannin identification.

Phlorotannins are formed by largely uncharacterized biosynthetic pathways, for which no restriction regarding bonding sites and macromolecular structure are known. Thus, for compounds with more than two PGUs, several possible structures arise, and their number increases drastically with the DP. Several phlorotannins have been described for a given DP, and their abundance in macroalgae, ecophysiological roles, and bioactivities have been shown to differ. Phlorotannins need to be identified at isomer-level, and that is largely achieved by NMR spectroscopy techniques. Unfortunately, NMR requires pure samples, at the milligram scale to be performed. An illustration of the ambiguity of most phlorotannin designators is present in Figure 6.

Mass spectrometry (coupled to HPLC) is an alternative method to reliably identify compounds in complex mixtures. Ionization on the ESI chamber is dependent on several factors, among which the pH of the media and the voltage applied. Depending on these, positive or negative ions will be predominantly formed and selected to enter the analyzer. Interestingly, while more is known about positive ionization (i.e., protonation, easily aided by weak acids), negative ionization (loss of acidic protons, and negative charge build-up), which is more easy to obtain in many different types of molecules, remains less mastered [204]. For instance, formic and acetic acids have often been used in macroalgae phenolic compounds elution and negative mode ionization [9,17,34,63,72,146,201,203]. Although acids have been known to promote positive mode ionization (through protonation), even aiming to improve chromatographic separation, avoiding tailing of the chromatographic peaks, its presence does not suppress the deprotonation at ESI source. Thus, experimental optimization is the safest way to achieve successful ionization.

Phenolic compounds contain numerous hydroxyl groups, which are somewhat acidic. To obtain quasimolecular ions, phenolic compounds eluates are often ionized in negative mode ([M − H]^−^). Polyphenol anions have been reported to be more stable than their counterpart cations, which undergo fragmentation rather easily [205]. However, several studies have analyzed phenolic compounds, particularly phlorotannins, in positive ionization mode [106,152,198,199,200], or using both modes [35,57,69,77,88,96,193,197]. Ideally, both should be performed, since other factors than the target compounds can have an influence on ionization performance (traces of salts, for instance). MS fragmentations can occur in both modes: quasimolecular ions of phlorotannins will undergo neutral fragment losses.

Different modes of spectra acquisition have been reported. Typical mass spectrometry involves the detection of the molecular ion and its products; by selecting a precursor ion, tandem mass spectrometry (MS^n^) can reveal further details of that ion’s own structure. This type of mass spectra acquisition is often used with a full scan of the range of *m/z*, and the limitation is usually the upper limit of the instrument. Thus, when full scan mode is used, one might consider the voltage manipulation of the spray, the capillary, and the orifice-skimmer-lens path in such a way to promote the desired level of fragmentation and/or multiple charge accumulation. Whereas the identification of macroalgae simple phenolic compounds, such as phenolic acids or flavonoids, has been successfully done by MS [14,17,18,21,57,63,72,110,120], different strategies have been found to characterize phlorotannins. For instance, phlorotannins of increasing DP have been detected due to the occurrence of di- and trivalent ionization, that cuts *m/z* values by a factor of 2 or 3 [10,28,34,36,61,104,194,195]. Besides SIM (referred above), other acquisition approaches have been useful for phlorotannins analysis, namely multiple reaction monitoring (MRM), which allows to choose a pair of parent-product ions known to be specific of a given compound. When multiple compounds co-elute (for example, in the case of same-DP isomers of phlorotannins), this strategy allows the quantification of each of them separately, should a specific fragment be known for the target analyte. MRM studies of phlorotannins can be found in Table 9 [18,28,165,166,193]. Data-dependent acquisition (DDA), which allows to follow MS/MS product ions from specific precursor ions and/or respective intensity, was also already applied, although in a study involving the macroalga *Ascophyllum nodosum* only the DP degree of 6 putative phlorotannins was tentatively assigned [88], while in a study with *Fucus vesiculosus* only a single phlorotannin was identified [106].

In fact, major difficulties have been identified in the application of MS to phlorotannins, since their isomerization by multiple combinations of PGUs relative positions has little impact on mass spectra. Therefore, it often fails to attribute a chemical structure to the detected molecules. Actually, MS can easily provide a profile of DPs in a phlorotannins mixture, such as the case of the study of Vissers et al., which differentiate the type and DP of *Laminaria digitata* phlorotannins from fractions obtained by NP-flash chromatography with UHPLC coupled to ion trap spectrometry [44]. In fact, diverse analyzers have been successfully used in the tentative identification of phlorotanins, such as triple quadrupole (QqQ) [118], ion trap (IT) [9], or quadrupole-time-of-flight (QTOF) [61]. This high precision analyzers have been effective in the structural elucidation, allowing the identification of the type of linkage and DP degree of several phlorotannins from *Fucus vesiculosus* [59], or other *Fucus* spp. [58]. Besides, MS of isolated phlorotannins has been performed almost exclusively on their peracetylated derivatives [37,40,41,45,46,62,154,171,184,185,206,207], since high-energy ionization techniques were the only techniques available at the time of their structural description, and such ionization was impracticable in the native compounds due to degradation. Thus, most MS data supported by NMR-confirmed identification is of electron impact (EI) or fast-atom bombardment (FAB) of acetylated phlorotannins, and have little value for the development of phlorotannins HPLC-MS.

Evidently, for a framework to be developed regarding mass spectra patterns, diagnostic fragments, and standard methodologies, literature needs to be sufficient for secure relations to be established. Table 10 shows the data retrieved on the monomers and dimmers of phloroglucinol, including that of halogenated derivatives. Noticeably, despite the attractiveness of these molecules and the abundant references to them in biological extracts studies of macroalgae, a scarcity of spectral information has been reported with sufficient detail and background validation to allow a definitive theory to be developed. For instance, most mass spectra in the table correspond to those obtained during the first description of the compound [40,41,45,46,208,209,210,211,212]. This is a problem for several reasons: MS had been performed, in such cases, with the intention to confirm molecular mass more than structure (which was invariably determined by NMR); most data was obtained by EI and fast atom bombardment (FAB), which reduces the ability to infer ESI-MS useful information; most data was obtained for the peracetyl-derivatives of the compounds, which might influence the mass spectra drastically; in most cases, relative abundances of the fragment ions were not reported; for most compounds, only one report could be found, which is not enough to assure reproducibility and to assess variability associated to operational specificities. Mass spectrometry data of higher molecular weight phlorotannins has been found to be equally impaired [19,154,184,185].

Other mass spectrometry-based techniques have been used to identify macroalgae phenolic compounds. Maheswari et al. identified one coumarin and two flavones from a crude extract of the brown macroalga *Padina tetrastromatica* by gas chromatography-mass spectrometry (GC-MS), using an HP-5 (supplied by Agilent) (30 m × 0.25 mm, internal diameter with 0.25 µm) column [20]. However, the primer limitation of EI is that the sample has to be vaporized by heating; therefore, this technique does not allows the analysis of higher molecular weight as well as thermally labile components, such as phlorotanins.

MALDI-TOF-MS, is a particularly suitable technique for the analysis of larger oligomers, with *m/z* above the upper limit of ESI-MS. In a study regarding the phlorotannin fraction of *Sargassum wightii*, MALDI-TOF-MS was used to confirm the presence of dimers, trimers, and hexamers of phloroglucinol [189]. This technique has been also used in combination with U/HPLC-ESI-MS providing information about the size and isomeric variation of phlorotannins. MALDI-TOF-MS allowed to detect phlorotannins with a DP between 7 and 17 ([M + Na]^+^ from 890 to 3370) in fractions of *Laminaria digitata* after fractionation with NP-flash chromatography [44].

### 7.2. NMR Analysis of Isolated Phenolic Compounds from Macroalgae Extracts

Although has being a technique used to analyze crude extracts or fractions, in order to confirm the reliability of spectrophotometric analysis (as discussed above), NMR has been also used in the identification (or confirmation) of isolated phenolic compounds [21,27,156,176], as well as of isolated phlorotannins [32,33,43,45,46,60,62,73,101,115,116,153,154,177,178,179,184,189,207,215,216,217]. In fact, NMR analysis has allowed to identify different groups of isolated phlorotannins, in particular, phlorethols, fuhalols, fucols, fucophlorethols, and eckols [32,33,40,43,45,46,60,62,73,153,154,177,178,179,184,207,215,216,217].

Although ^1^H NMR and/or ^13^C NMR have been always used in macroalgae phenolic compounds identification, some authors have complemented their analysis with two-dimensional NMR (2D NMR), namely. heteronuclear multiple-quantum correlation (HMQC), heteronuclear multiple-bond correlation (HMBC), ^1^H-^1^H rotating frame nuclear overhauser effect spectroscopy (ROESY), or ^1^H-^1^H correlation spectroscopy (COSY) [32,40,62,73,115,177,178,184,207].

## 8. Conclusions

In this review the subject of macroalgae phenolic compounds extraction and characterization is tackled since it has been identified as a very important piece of the biotechnological valorization of macroalgae, for which a big picture was lacking, and technical challenges were being overlooked. In this context, a critical analysis of the state-of-the-art, accompanied by a compilation of parameters and results published on macroalgae phenolic extracts profile, extraction, and analysis has been conducted, resulting in (a) a systematization of consensual methodologies, of misconceptions, and current trends in phenolic compounds macroalgae research; and (b) the identification of the most effective extraction, fractionation, and characterization techniques and conditions.

Essentially, extraction methodologies were found to be the root of most problems associated with the slower-than-expected valorization of these components. Currently, aqueous mixtures of alcohol and acetone are consensually being used in the extraction of macroalgae phenolic compounds. Nevertheless, they result in extracts of high complexity, with quite abundant polysaccharides and other metabolites, impairing the direct isolation and characterization of novel phenolic compounds from macroalgae (e.g., HPLC-MS). Besides environmental concerns, several novel and alternative extraction methodologies have already been studied for the extraction of phenolic compounds from macroalgae. However, the lack of extract characterization does not allow in-depth assumptions, particularly regarding the selectivity of these techniques.

Even from an environmental and sustainability point of view, the solvent composition that has been used to extract macroalgae phenolic compounds is not compatible with industrial volumes of biomass processing due to environmental and financial reasons.

Further, methods to fractionate these extracts and obtain phenolic compounds-enriched fractions are still insufficient in preparing the extracts for analytical platforms. It is thus of paramount importance to develop novel extraction and primary fractionation methodologies that potentiate both the generation of solid scientific data by researchers, and, ultimately, the industrial exploration of macroalgae biomass. In an analytical perspective, sequential fractionation of crude macroalgae extracts have already been efficaciously developed for the isolation of phenolic compounds; notwithstanding, less time-consuming liquid–liquid extraction with n-hexane followed by partitioning with ethyl acetate was shown to be a suitable technique to obtain macroalgae phenolic compounds-rich fractions able to be characterized by U/HPLC-ESI-MS.

Although the identification of phenolic compounds on macroalgae has increased, which has skyrocketed the macroalgae phenolic compounds research, there is still a lack of information on the structural identification of higher molecular weight compounds, particularly in the case of phlorotannins. In addition, methodologies that are able to quantify such components are also missing. However, it is anticipated that this review will decrease the number of studies focused only on spectrophotometric analysis.

On this vein, it is expected that this appraisal will be an important basis for the in years to come exploitation of phenolic fraction of macroalgae allowing the use of the most profitable methodologies of extraction, fractionation, and characterization, as well as giving the priority-challenges to be overcome for a macroalgae bio-industry developed on the basis of macroalgae phenolic compounds.

## Figures and Tables

**Table 1:** Solvent mixture, SLr, extraction time, and temperature employed for conventional solid–liquid extraction (SLE) of phenolic compounds from macroalgae ^a^.

Solvent Mixture	SLr (*w/v*)	t (h)	T (°C)	Species	Phenolic Compounds Extracted	Type of Characterization ^b^	Reference
Ethyl acetate, acetone, ethanol, methanol, acetone:H_2_O (70:30), ethanol:H_2_O (80:20), methanol:H_2_O (80:20), H_2_O	1:50	1 (×4)	r.t.	*Fucus vesiculosus*	n.i. ^d^	TPC	[79]
Acetone:water (70:30) + 0.3% ascorbic acid	1:50	1 (×4)	r.t.	*Fucus vesiculosus*	Phlorotannins	TPC, HPLC-MS	[34]
Ethanol	1:1.6	2	On ice	*Fucus vesiculosus*	Phlorotannins	HPLC-DAD, HPLC-ESI-MS, UV–vis, FT-IR, NMR	[62]
Methanol, acetonitrile:water (50:50) + 0.2% formic acid	1:10	overnight	r.t.	*Ascophyllum nodosum*, *Ulva lactuca*, *Palmaria palmata*, *Alaria esculenta*	Phlorotannins	TPC, AA, HPLC-MS	[77]
Acetone:water (70:30)	1:20	1 (×4)	r.t.	*Cystoseira nodicaulis*, *Cystoseira tamariscifolia*, *Cystoseira usneoides*, *Fucus spiralis*	Phlorotannins	AA, HPLC-MS	[35]
Acetone:water (70:30)	1:20	1 (×4)	r.t.	*Cladostephus spongiosus*, *Cystoseira nodicaulis*, *Cystoseira tamariscifolia*, *Cystoseira usneoides*, *Fucus spiralis*, *Halopteris filicina*, *Padina pavonica*, *Saccorhiza polyschides*, *Sargassum vulgare*, *Stypocaulon scoparium*	n.i.	TPhC	[70]
Methanol:water (80:20)	1:7.5–13.2 followed by 1:3.4–6.6	0.5 + 3 (×3)	r.t.	*Fucus vesiculosus*, *Pelvetia canaliculata*, *Fucus spiralis*, *Ascophyllum nodosum*, *Saccharina longicruris*	Phlorotannins	HPLC-MS	[10]
Methanol: water (80:20), ethyl acetate: water (80:20), acetone: water (70:30), ethanol:water (80:20 and 50:50)	1:25	24	r.t.	*Fucus vesiculosus*	Phlorotannins	TPC, AA, HPLC-MS	[80]
Water	1:20	24	r.t.	*Ascophyllum nodosum*, *Pelvetia canaliculata*, *Fucus spiralis*, *Ulva intestinalis*	n.i.	TPC, AA	[87]
Ethanol:water (80:20)	1:10						
Acetone:water (80:20)	1:10						
Ethanol:water	1:10	24	r.t.	*Fucus spiralis*, *Pelvetica canaliculata*, *Ascophyllum nodosum*	Phlorotannins	TPC, AA, MS	[78]
Water	1:20	24	r.t.				
Water	1:20	24	60				
Ethanol	1:10	12	r.t.	*Eisenia bicyclis*	Phlorotannins	HPLC-DAD	[38]
Water, water + HCl 5 mM, water + 0.2% formic acid, acetonitrile: water (50:50) + 0.2% formic acid	1:10	1	r.t.	*Ascophyllum nodosum*	Phlorotannins	HPLC-MS	[88]
Water	1:100	5 min	100	*Ecklonia cava*, *Ecklonia stolonifera*, *Eisenia bicyclis*	Phlorotannins	AA, HPLC-UV, NMR	[86]
Methanol	1:4	2	r.t.				
Ethanol	1:10	overnight	r.t.	*Fucus serratus*, *Fucus vesiculosus*, *Fucus distichus*, *Fucus spiralis*, *Sargassum muticum*, *Saccharina latissima*, *Laminaria digitata*, *Dictyota dichotoma*, *Enteromorpha intestinalis*, *Ulva lactuca*, *Palmaria palmata*, *Porphyra purpurea*, *Chondrus crispus*, *Mastocarpus stellatus*, *Polysiphonia fucoides*, *Gracilaria vermiculophylla*	Phenolic acids	TPC, AA, HPLC-DAD	[12]
Water	1:20						
Methanol:water (80:20)	1:10	2	r.t.	*Kappaphycus alvarezii*	n.i.	TPC, TFC, TAC, AA	[66]
Ethanol:water (80:20)	1:10	24	r.t.	*Fucus*, *serratus*, *Fucus vesiculosus*, *Himanthalia elongata*, *Cystoseira nodicaulis*	Phlorotannins	TPC, AA, HPLC-MS	[28]
Water	1:15	4	55	*Macrocystis pyrifera*	Phlorotannins	TPC, AA, HPLC-MS	[69]
Methanol:water (60:40)	1:10	2	40	*Himanthalia elongata*	Phloroglucinol, phenolic acids, flavonoids and phenolic terpenes	TPC, AA, HPLC-DAD-MS	[17]
H_2_O, acetone, ethanol	1:2	19	r.t.	*Fucus vesiculosus*	Phlorotannins	TPC, UV–vis, FT-IR, NMR	[91]
Methanol:water (80:20)	1:40	1.5	20	*Laminaria digitata*	Phlorotannins	TPC, TPhC, NMR, UHPLC-UV-MS, MALDI-TOF-MS	[44]
Acetone	-	-	- ^e^	*Padina pavonica*	n.i.	AA, TPC, TFC, TTC	[92]
Ethanol:water (80:20)	1:20	24	r.t.	*Fucus vesiculosus*	Phlorotannins	UHPLC-DAD-MS	[59]
Ethanol	1:1.4	30 days	r.t.	*Costaria costata*, *Fucus evanescens*	Phlorotannins	NMR, UV–vis, ESI-MS	[61]
Methanol	1:10	72	r.t.	*Padina tetrastromatica*	Coumarin and flavones	TPC, AA, GC-MS	[20]
Ethanol:water (80:20)	-	2 × 72	55	*Ecklonia cava*	Phlorotannins	NMR	[101]
Methanol:water (70:30)	1:50	-	-	*Caulerpa spp.*	n.i.	TPC, TFC, AA	[102]
Ethyl acetate	1:10	6	60–80 ^c^	*Sargassum wightii*	Phlorotannins	AA, FT-IR, HPLC-MS	[56]
Ethanol:water(20:80, 40:60, 60:40, 80:20, 100:0)	1:25	12	r.t.	*Cladophora glomerata*	Phenolic acids and flavonoids	TPC, AA, HPLC-DAD-MS	[14]
Methanol:water (60:40)	1:10	2	40	*Himanthalia elongata*	Phenolic acids and flavonoids	TPC, AA, HPLC-DAD-ESI-MS	[63]
				*Laminaria digitata*, *Laminaria saccharina*, *Himamthalia elongata*	n.i.	TPC, TFC, TTC, AA	[63]
Ethanol:water (50:50)	1:10	24	25–35	*Laurencia caspica*	n.i.	TPC, AA	[89]
Acetone:water (70:30)	1:70	3	25	*Fucus vesiculosus*	Phlorotannins	TPhC, UHPLC-DAD-ESI-MS	[9]
Ethanol:water (50:50)	1:2.5	24	r.t.	*Durvillaea antarctica*, *Lessonia spicata*, *Macrocystis integrifolia*	Miscellaneous phenolic compounds	TPC, AA, HPLC-UV-ESI-MS	[19]

^a^ For those involving optimization, the optimal conditions are shown. ^b^ TPC—total phenolic content; TPhC—total phlorotannins content; TFC—total flavonoids content; TAC—total anthocyanin content; TTC—total tannins content; AA—antioxidant activity; HPLC-DAD—high performance liquid chromatography with photodiode array detector; UHPLC—ultra high-performance liquid chromatography; ESI-MS—electrospray ionization-mass spectrometry; NMR—nuclear magnetic resonance; UV—ultraviolet spectroscopy; FT-IR—Fourier-transform infrared spectroscopy; GC-MS—gas chromatography-mass spectrometry; MALDI-TOF-MS—matrix-assisted laser desorption/ionization time-of-flight mass spectrometry. ^c^ r.t.—room temperature. ^d^ n.i.—compounds not identified. ^e^ Soxhlet extraction.

**Table 2:** Conditions of EAE applied to the extraction of phenolic compounds from macroalgae.

Macroalgae	Type of Enzyme	t (h)/pH/T (°C)	Phenolic Compounds Extracted	Type of Characterization ^a^	Extraction Methodology Associated	Reference
*Hizikia fusiformis*	Endo-peptidase + β-glucanase	24/7.0–8.0/50–60	n.i.^b^	TPC, AA	Conventional SLE	[99]
*Palmaria palmata*	Endo- and exo-peptidase complex	24/7.0/50	n.i.	TPC, AA	-	[97]
	Endo-peptidase	24/8.0/50				
	Protease complex	24/6.0/40				
	Amino- and carboxy-peptidase	24/6.0/40				
	Metallo-endo-protease	24/6.0/50				
	Endo-protease and exo-peptidase	24/7.0/50				
	Multi-enzyme complex	24/4.5/50				
	β-Glucanase	24/7.0/60				
	Exo-1,4-α-glucosidase	24/4.5/60				
	Cellulase	24/4.5/50				
	α-Amylase	24/6.0/60				
*Sargassum muticum*, *Osmundea pinnatifida*, *Codium tomentosum*	Endo-peptidase	24/8.0/50	Flavan-3-ols	HPLC-UV-ESI-MS	-	[95]
	Endo-protease and exo-peptidase	24/7.0/50				
	Cellulase	24/4.5/50				
	Multi-enzyme complex	24/4.5/50				
*Lessonia nigrescens*, *Macrocystis pyrifera*, *Durvillaea antarctica*	Cellulase	17/4.5/50	n.i.	TPC	-	[98]
	α-Amylase	17/6.0/60				
*Ecklonia radiata*	Multi-enzyme complex	24/4.5/50	n.i.	TPC, AA	Microwave-assisted enzymatic extraction	[100]
	Cellulase	24/4.5/50				
	β-Glucanase	24/7.0/50				
	Endo- peptidase	24/8.0/50				
	Metallo-endo-protease	24/6.0/50				
	Endo-protease and exo-peptidase	24/7.0/50				
*Sargassum muticum*	Endo-peptidase	2 and 4/7.0/50	Hydroxytetrafuhalol, triphlorethol and dihydroxypentafuhalol	TPC, TPhC, AA, HPLC-DAD-MS	ASE applied to the biomass residue	[96]
	Multi-enzyme complex	2 and 4/4.5/50				
*Macrocystis pyrifera*	Alginate lyase + fucoidanase + 1,3-β-d-glucanase	36/7.0/25	Phloroeckol and 1 tetramer of phloroglucinol	TPC, AA, HPLC-UV-ESI-MS	Conventional SLE	[71]

^a^ TPC—total phenolic content; AA—antioxidant activity; TPhC—total phlorotannins content; HPLC—high performance liquid chromatography; UV—ultraviolet detector; DAD—photodiode array detector; ESI—electrospray ionization; MS—mass spectrometry. ^b^ n.i.—compounds not identified.

**Table 3:** Accelerated solvent extraction (ASE) conditions applied to the extraction of phenolic compounds from macroalgae.

Solvent of Extraction (*v*:*v*)	P (psi)	T (°C)	t (min)	Macroalgae Species	Compounds Extracted	Type of Characterization ^a^	Reference
Ethanol:water (25:75; 75:25)	1500	120	20	*Sargassum muticum*	n.i. ^b^	TPC, AA, NMR	[8]
Water	1500	120	25	*Fucus serratus*, *Laminaria digitata*, *Gracilaria gracilis*, *Codium fragile*	n.i.	TPC, AA	[90]
Ethanol:water (80:20)	1000	100					
Methanol:water (70:30)	1000	90					
Ethanol:water (95:5)	1500	160	20	*Sargassum muticum*	Phlorotannins	TPC, TPhC, AA, LCxLC-DAD-MS	[104]
Ethanol:water (72:25)	1500	120	20	*Sargassum muticum*	Phlorotannins	TPC, TPhC, AA, HPLC-DAD-MS	[96]
Dichloromethane + Methanol ^b^	-	-	5 + 5 ^c^	*Fucus vesiculosus*	Phlorotannins	AA, HPLC-DAD-MS	[106]
Hexane, ethyl acetate, ethanol, ethanol:water (50:50)	1500	80, 120, 160	10	*Laminaria ochroleuca*	n.i.	TPC	[105]

^a^ TPC—total phenolic content; TPhC—total phlorotannins content; AA—antioxidant activity; NMR—nuclear magnetic resonance; HPLC—high-performance liquid chromatography; DAD—photodiode array detector; MS—mass spectrometry; LCxLC—two-dimensional liquid chromatography. ^b^ n.i.—compounds not identified. ^c^ dichloromethane followed by methanol extraction—extracts were combined for characterization.

**Table 4:** Microwave-assisted extraction (MAE) conditions applied to the extraction of phenolic compounds from macroalgae.

Extraction Solvent	SLr	t (min)	T (°C)/Power (w)	Macroalgae	Phenolic Compounds Extracted	Type of Characterization ^a^	Reference
Ethanol:water (20–100%)	1:10–1:50	5–60	20–70/100–600	*Caulerpa racemosa*	n.i. ^b^	TPC, AA, FT-IR, HPLC-UV	[109]
Ethanol:water (50:50; 60:40; 70:30)	1:4–1:12	5–25	45–65/400–600	*Saccharina japonica*	n.i.	TPC	[108]
Solvent free followed by water addition	-	5–35 (×1–5)	-/200–900	*Sargassum muticum*	n.i.	TPC, AA, HPLC-DAD	[112]
Ethanol	-	10 × 2	-/400				
Ethanol:water (50:50)	-	10 × 3	-/400				
Water ^c^	-	180	50/-	*Ecklonia radiata*	n.i.	TPC, AA	[100]
Water	1:3	30	25,40,60/1000	*Polysiphonia*, *Ulva* and *Cladophora* (mixed)	n.i.	TPC	[111]
Acetone:water (100:0; 30:70; 50:50; 70:30; 0:100)	1:20	4–12	-/200–600	*Chaetomorpha sp*.	n.i.	TPC, AA	[54]
Methanol:water (70:30)	1:10	15	110/-	*Ascophyllum nodosum*, *Laminaria japonica*, *Lessonia trabeculate*, *Lessonia nigrecens*	Phenolic acid, flavonoids, phlorotannins	TPC, AA, HPLC-DAD-MS	[110]

^a^ TPC—total phenolic content; AA—antioxidant activity; FT-IR—Fourier-transform infrared spectroscopy; HPLC—high-performance liquid chromatography; DAD—photodiode array detector; UV—ultraviolet detector; MS—mass spectrometry. ^b^ n.i.—compounds not identified. ^c^ Microwave-assisted enzymatic extraction.

**Table 5:** Ultrasound-assisted extraction (UAE) conditions applied to the extraction of phenolic compounds from macroalgae.

Extraction Solvent	Frequency/Power (kHz/W)	Conditions	Macroalgae	Phenolic Compounds Extracted	Type of Characterization ^a^	Reference
Water and water + HCl (0.03M)	20/750	10 min; amplitude between 22.8 and 114 µm	*Ascophyllum nodosum*	n.i. ^b^	TPC	[114]
Water + HCl (0–0.06 M)	20/750	5–25 min; amplitude between 22.8 and 114 µm	*Ascophyllum nodosum*	Phlorotannins	TPC, ESI-MS	[55]
Water	50–60/400	24h ^c^ + 60 min (pauses of 2 min after each 10 min of sonication); 50 °C	*Sargassum muticum*, *Osmundea pinnatifida* and *Codium tomentosum*	n.i.	TPC, AA	[95]
Ethanol	40/250	30–60 min; 30–50°C	*Laurencia obtusa*	n.i.	TPC, AA	[117]
Water	-/-	5 min^c^ + 10 min	*Ascophyllum nodosum*, *Bifurcaria bifurcata*, *Fucus vesiculosus*	Phenolic acids, flavonoids and phlorotannins	HPLC-DAD-ESI-MS	[118]
Methanol	-/-	5 × 15 min	*Dasycladus vermicularis*	Coumarins	ESI-MS, ^1^H NMR, ^13^C-NMR	[21]
Methanol:water (50:50)				Coumarins and phenolic acids		
Ethanol:water (50:50)	-/-	30 min, r.t. ^d^ +2 h, 70 °C ^c^	*Macrocystis pyrifera*, *Ecklonia arborea*, *Silvetia compressa*, *Ulva intestinalis*, *Solieria filiformis*	Flavonoid glycosides and phlorotannins	TPC, HPLC-ESI-MS	[119]
Ethanol:water (55:45)	45/-	90 min, 60 °C	*Enteromorpha prolifera*	Naphtalenone, flavone and flavanone glycosides	UHPLC-ESI-MS	[120]
Methanol:water (50:50)	-/-	1 h + 24 h ^c^ + 1 h r.t.	*Fucus guiryi*, *Fucus serratus*, *Fucus spiralis*, *Fucus vesiculosus*	Phlorotannins	TPhC, HPLC-ESI-MS	[58]
Acetone:water (70:30)	35/-	5.75 h, 30 °C	*Agarum cribrosum*	Trifuhalol	TPC, AA, HPLC-DAD-ESI-MS	[60]
Methanol:water (80:20)	-/-	2 days, r.t.	*Ecklonia cava*	Phlorotannins	^1^H NMR, ^13^C-NMR, FT-IR, HPLC-ESI-MS	[116]
**Methanol**	-/-	2 h, 20 °C	*Ulva intestinalis*, *Codium tomentosum*, *Dictyota dichotoma*, *Halopteris scoparia*	Flavone	TPC,	[57]
Ethanol:water (80:20)	-/-	90 min;	*Ecklonia maxima*	Eckmaxol	HR-ESI UHPLC-ESI-MS-MS, ^1^H NMR, ^13^C-NMR	[115]

^a^ TPC—total phenolic content; AA—antioxidant activity; TPhC—total phlorotannins content; ESI-MS—electrospray ionization-mass spectrometry; HR—high resolution; ^1^H NMR—proton nuclear magnetic resonance; ^13^C-NMR—carbon nuclear magnetic resonance; HPLC—high-performance liquid chromatography; UHPLC—ultra-high-performance liquid chromatography; DAD—diode array detector; FT-IR—Fourier-transform infrared spectroscopy. ^b^ n.i.—compounds not identified. ^c^ conventional maceration. ^d^ r.t.—room temperature.

**Table 6:** Supercritical carbon dioxide (SC-CO_2_) supercritical fluid extraction (SFE) conditions applied to the extraction of phenolic compounds from macroalgae.

SFE Modifier	Pressure (MPa)/Temperature (°C)/FlowCO_2_ (kg CO_2_ h^−1^)	Extraction Time (h:min)	Macroalgae	Phenolic Compounds Extracted	Type of Characterization ^a^	Reference
Ethanol (3%)	8–30/30–60/1.69	1:00	*Undaria pinnatifida*	n.i. ^b^	TPC	[126]
Methanol:water (10:90) (3%)	35/40/-	1:00	*Sargassum muticum*, *Sargassum vulgare*, *Hypnea spinella*, *Porphyra sp.*, *Undaria pinnatifida*, *Chondrus crispus* and *Halopytis incurvus* (mixed)	Isoflavones	Fast LC-MS	[18]
Ethanol (12%)	15.2/60/-	1:30	*Sargassum muticum*	n.i.	TPC, AA, NMR	[8]
Ethanol (0.5–10%)	10–30/30–50/1.5	1:00	*Sargassum muticum*	n.i.	TPC, AA	[125]
-	50/40/200	5:17–13:28	*Cladophora glomerata*, *Ulva flexuosa* and *Ulva clathrata* (mixed)	n.i.	TPC	[127]
-	37.9/50/34	-	*Laminaria digitata*, *Undaria pinnatifida*, *Porphyra umbilicalis*, *Eucheuma denticulatum*, *Gelidium pusillum*	Phlorotannins^c^	HPLC-DAD	[42]

^a^ TPC—total phenolic content; AA—antioxidant activity; LC—liquid chromatography; MS—mass spectrometry; NMR—nuclear magnetic resonance; HPLC-DAD—high-pressure liquid chromatography-mass spectrometry. ^b^ compounds not identified. ^c^ only qualitatively identified by a computer-driven algorithm.

**Table 7:** Column chromatography conditions reported in macroalgae polar extractives fractionation.

Compounds	Stationary Phase	Mobile Phase ^a^	Reference
**Normal-Phase**			
**Phlorotannins**	Silica gel	TCM:MeOH 9:1	[45]
		TCM to TCM:MeOH 4:1	[46]
		Hex to Hex:EtOAc 1:1 to DCM to DCM:MeOH 1:1	[153]
		DCM:MeOH 6:1–1:6	[169]
		TCM:MeOH 99:1 to MeOH	[143]
		TCM:Hex 1:1–4:1, TCM-AcetO 49:1–4:1, MeOH	[43,154,162,170,171]
		EtOAc–MeOH 50:1–5:1	[32,76,151,172]
		DCM, MeOH	[173]
		TCM:MeOH 1:1	[174]
		TCM:MeOH 100:1 to 1:1	[157]
		EtOAc:MeOH 50:1−5:1	[32]
		EtOAc, MeOH, Water	[148]
		TCM:MeOH:Water 80:18:2–50:49:1	[175]
		Hex:EtOAc 1:1	[32,76]
		Hex to AcetO to MeOH	[44]
		Benz to Benz:EtOAc(10:1–1:1) to EtOAc to TCM to TCM:EtOH(30:1–1:1) to EtOH	[61]
		Hex:EtOAc 9.5:0.5–7.5:2.5	[58]
	Diatomaceous earth	Hex, DCM, EtOEt, MeOH	[152]
**Miscellaneous**	Silica gel	EtOAc:MeOH 1:0 to 0:1	[17]
**Phenolic acids**	Silica gel	Hex–TCM 1:1, TCM:MeOH 1:1	[13]
**Flavones**	Silica gel	TCM:MeOH 3:2	[176]
**Aurones**	Silica gel	Hex:TCM:MeOH 1:0:0–0:1:0–0:0:1	[23]
**Coumarins**	Silica gel	DCM, EtOAc, MeOH, water	[21]
**Reversed-Phase**			
**Phlorotannins**	Octadecyl (unspecified)	MeOH (aq.) 10–100%	[177]
		MeOH (aq.) 20–100%	[32,76,151]
	Cosmosil 75C 18-OPN	MeOH (aq.) 0–100%	[159]
	LiChroprep RP-18	MeOH (aq.) 20–100%	[172,178]
**Coumarins and phenolic acids**	Reveleris RP-18	MeOH (aq.), 0.25% FA	[21]
**Size-Exclusion**			
**Phlorotannins**	Sephadex LH-20	MeOH	[76,116,151,174,175,177,178,179,180]
		EtOH (aq.) 50–80%, AcetO (aq.) 50–80%	[77]
		MeOH (aq.) 80%	[157]
		MeOH (aq.) 60–100%, AcetO (aq.) 70%	[181]
		MeOH:EtOAc (7:3)	[156]
		TCM:MeOH 2:1–1:1–0:1	[182]
		EtOH (aq.) 50–0%, AcetO (aq.) 0–70%	[88]
		MeOH:water 1:1–1:3–0:1, MeOH:AcetO 5:1–3:1–1:1	[60]
		DCM:MeOH (1:1)	[115]
**Coumarins**	Sephadex LH-20	MeOH (aq.) 50%	[21]

^a^ TCM—chloroform; DCM—dichloromethane; MeOH—methanol; EtOH—ethanol; EtOAc—ethyl acetate; EtOEt—diethyl ether; Hex—hexane; AcetO—acetone; FA—formic acid; aq.—aqueous.

**Table 8:** Preparative high-performance liquid chromatography (HPLC) conditions for the isolation of phenolic compounds from macroalgae.

Column (a/b/c) ^a^	Mobile Phase ^b^	Flow-Rate (ml/min)	UV Detection (nm)	*Species* Outcome [Reference]
**Normal Phase**				
**LiChrosorb Si-60** **(250/16/7)** **(250/8/5)**	A: TCM B: EtOH B: 0.5–3.0%	-	275	*Sargassum spinuligerum* Isol. of 20 fuhalols and deshydroxufuhalols [154].
	A: TCM B: EtOH B: 0–2.5%	-	275	*Sargassum spinuligerum* Sep. of pseudofuhalols [170] Isol. of 14 new (hydroxy)phlorethols and [(des)hydroxy]fuhalols [171].
				*Sargassum spinuligerum* and *Cystophora torulosa.* Isol. of 9 new fucophlorethols and 8 known ones from NP-CC fractions [43].
				*Cystophora retroflexa* Isol. of 18 phlorethols and fucophlorethols (to isomer lever) [184] Isol. of 12 halogenated phlorethols and fucophlorethols (partially co-eluted) [41].
**LiChrosorb Si-60** **(250/16/7)**	TCM-Hex (+0.3% MeOH) TCM-MeOH (higher MW)	-	-	*Cystophora congesta* Isol. of 11 brominated and non-halogenated phlorotannins [46].
**LiChrosorb Si-60** **(250/8/5)**	TCM-Hex TCM-MeOH (higher MW).	-	250–275	*Carpophyllum angustifolium* Isol. of 3 trihidroxyphlorethols (2 isomers) [40] Sep. of NP-CC fractions into 4 trihidroxyphlorethols [185].
**LiChrosorb Si-60** **(250/7.5/10)** **Partisil 10** **(500/5/10)**	A: TCM B: EtOH B: 0.3–2.0%	-	275	*Eisenia arborea.* Sep. of extract into 17 fractions, containing a total of 21 compounds (19 new eckols) [45].
**Pharmprep60CC SiO_2_** **(250/2.1/-)**	0.1% AcAc (aq.)	5.0	-	*Fucus vesiculosus* Enrichment of crude extract to further preparative RP-HPLC [91].
**LiChrosorb Si-60** **(250/10/5)**	A: TCM:water (5:95) B: EtOH %B: 15–100	1.0	270	*Laminaria digitata*, *Undaria pinnatifida*, *Porphyra umbilicalis*, *Eucheuma denticulatum*, *Gelidium pusillum* [42].
**Reversed-Phase**				
**µBondapak C18** **(300/7.8/10)**	36% ACN	1.0	245	*Ecklonia cava* Isolation of dieckol (Rt~6 min) from NP-CC fraction [173].
**Luna-C18** **(-)**	A: 0.1% FA (aq.) B: 0.1% FA in ACN 20–100% B	3.0	245	*Ecklonia stolonifera* Isolation of 2-phloroeckol, eckol, phlorofucofuroeckol B and bieckol from previous RP-HPLC fractions [32].
**Shim-pack PREP-ODS** **(250/20/5)**	A: 0.1% FA (aq.) B: 0.1% FA in ACN 20–100% B	7.0	245	*Ecklonia stolonifera* Separation of SEC-HPLC fraction into 6 subfractions. SF1, SF4, SF5, SF6 further purified by RP-HPLC [32,169].
**Develosil ODS5** **(-)**	A: 0.1% TFA (aq.) B: 0.1% TFA in ACN 0–30% B	-	-	*Eisenia arborea* Isolation of active fraction at TR 130–150 min; Repetition with 47% methanol 0.1% TFA for isolation of phlorofucofuroeckol B at tR = 41 min [73].
**C18** **(150/4.5/5)**	18% ACN (aq.) (+0.1% formic acid)	0.7	-	*Ecklonia maxima* Isolation of eckol, 7-phloroecckol and 2-phloroeckol [186].
**J’sphere ODS-H80** **(150/20/4)**	MeOH (aq., increasing % *v/v*)	0.8	230	*Ecklonia cava* Purification of dieckol with purity > 95% from SEC-CC fraction [157,182].
**Alltima C18 column** **(250/10/5)**	MeOH 30–100%	4.0	290	*Ecklonia cava* Purification of dieckol and phlorofucofuroeckol A from SEC-CC fraction [180].
**Mightysil RP-18 GP II (250/10/5)**	MeOH 40%(1.0% FA aq.)	0.5	DAD 190–500 (rec = 204–208)	*Ecklonia kurome* Separation of dieckol, 974-B, 974-A, phlorofucofuroeckol B and phlorofucofuroeckol A from RP-CC fraction [159].
**C18** **(250/10/-)**	30–100% MeOH	1.0	290	*Ecklonia cava* Isolation of 7-phloroeckol and eckol from ethyl ether fraction of crude extract [187]. Isolation of dieckol, phlorotannin 974B, phlorotannin 974A, and phlorofucofuroeckol-A from ethyl ether fraction of crude extract [188].
**Phenomenex aqua C18 (250/10/5)**	A: 1% AcAc B: 1% AcAc in ACN %B: 1–100%	2.5	-	*Fucus vesiculosus* 10 fractions. Repetition with isocratic A:B 90:10 isolated six subfractions, from which 3 fucophlorethols were isolated [62].
**Amberlite XAD7HP** **(250/21/-)**	A: 0.1% AcAc (aq.) B: ACN %B: 0–100%	5.0	-	*Fucus vesiculosus* *67 fractions. Bioassay-guided fractionation* [91].
**LiChrosorb RP-18** **(250/8/5)** **(250/10/7)**	0.015 M K_2_SO_4_	-	235	*Pleurophycus gardneri* Isolation of sulphated phlorotanin from PVP adsorbate of crude extract [39].
	2 to 50% MeOH (aq.)	1.0	350	*Dasycladus vermicularis* Isolationf of a sulphated coumarin from a RP-CC fraction [21].
**C18** **(50/2.1/1.8)**	A: 0.5% TFA (aq.) B: 0.5% TFA in ACN %B: +4%/min	1.0	280	*Sargassum wightii*, *S. tenerrimum and Turbinaria conoides* Isolation of a phlorotannins-enriched fraction from crude extract [189].
**Optimapack C18** **(250/10/10)**	A: ACN (aq.)/0.1% FA B: ACN %B: 20 (65 min)–100 (65 min)/30 (60 min)–100 (65 min)	2	-	*Ecklonia cava* Isolation of phlorofucofuroeckol, dioxinodehydroeckol and fucofuroeckol [116].
**Size-Exclusion**				
**Asahipak GS-310** **(500/20/13)**	MeOH	5.0	245	*Ecklonia stolonifera* Separation of NP-CC fraction in 5 subfractions; SF4 further purified by RP-HPLC [32,169].
**Superose 6 colum** **(300/10/13)**	100 mM sodium acetate buffer (pH 6.3) 20 mM EDTA, 250 mM NaCl	0.25	210	*Padina gymnospora* Recovery of 2-[1′-Oxo-hexadecyl]-1,3,5-trihydroxybenzene (a phloroglucinol derivative) from RP-CC fraction [164].

^a^ letters *a*, *b* and *c* are for length, in millimeters, internal diameter, in millimeters, and particle size, in micrometers. ^b^ TCM—chloroform; MeOH—methanol; EtOH—ethanol; Hex—hexane; ACN—acetonitrile; TFA—trifluoroacetic acid; aq.—aqueous; FA—formic acid; AcAc—acetic acid.

**Table 9:** HPLC-mass spectrometry (MS) conditions for the on-line analysis of macroalgae phenolic compounds during chromatographic separation.

Species [Reference]	Outcome	Configuration ^a^	MS Mode ^b^	HPLC Column	Mobile Phase ^c^
*Fucus vesiculosus* [34]	Detection of 16 phlorotanins.	NP-HPLC-ESI-QqQ	(-) FULL SCAN	LiChrospher Si 60 250 × 4 mm, 5 µm LiChrospher Si 60 guard cartridge 4 × 4 mm, 5 µm	A: 2% water, 2% AcAc, 14% MeOH in DCM B: 2% water, 2% AcAc in MeOH %B: 0–87.8 (50 min), 87.5−0 (80 min) 1.0 mL/min
*Sargassum muticum*, *Sargassum vulgare*, *Hypnea spinella*, *Porphyra* sp., *Undaria pinnatifida*, *Chondrus crispus*, *Halopytis incurvus* [18]	Sep. and ident. of 8 isoflavones	NP-HPLC-ESI-QqQ	(-) MRM	Zorbax SB-CN 100 × 2.1 mm, 3.5 μm	A: 0.2% AcAc (aq.) B: ACN %B: 30–80 (6 min), 80−30 (10 min) 0.4 mL/min
*Fucus distichus*, *Saccharina latissima*, *Saccharina groenlandica*, *Alaria marginata*, *Porphyra fallax*, *Ulva lactuca*, [197]	Sep. and ident. of 24 phlorotannins (DP-level)	SEC-HPLC-ESI-IT-TOF	(+)/(-) FULL SCAN	Develosil Diol 250 × 4.6 mm, 5 μm	A: 0.2% AcAc in ACN B: 0.2% AcAc, 3% water in MeOH %B: 0–100 (40 min), 100–0 (50 min) 0.8 mL/min
*Ecklonia stolonifera* [198]	Sep. and tentative ident. of 18 phlorotannins	RP-HPLC-ESI-IT	(+) FULL SCAN	Capcell Pak C18 UG120 2.0 × 150 mm, -	A: 1% FA in MeOH:water (5:95) B: 1% FA in MeOH:water (70:30) %B: 0–100 (60 min) 0.2 mL/min
*Ecklonia stolonifera* [199]	Separation and identification of 3 phlorotanins.	RP-HPLC-ESI-IT	(+) FULL SCAN	Hypersil Gold C-18 250 × 4.6 mm, 5 µm Gemini C-18 guard column 30 × 4.6 mm, 5 µm	A: 0.1% FA (aq.) B: 0.1% FA (in ACN) %B: 13–60 (48 min),60−13 (60 min) 1.0 mL/min
*Porphyra tenera*, *Undaria pinnatifida* [72]	Sep., ident. and quant. of 12 misc. phenolics	RP-HPLC-ESI-Q	(-) SIM	Zorbax SB-C18 150 × 4.6 mm, 3.5 μm	A: 0.2% AcAc (aq.) B: ACN %B: 4–30 (10 min), 30−4 (15 min) 1.1 mL/min
*Porphyra dentata* [200]	Sep. and ident. of 1 catechol and 2 flavonoid glycosides	RP-HPLC-ESI-IT	(+) FULL SCAN	Luna C18 150 × 2 mm, 3 µm	A: 0.05% TFA in ACN B: 0.05% TFA (aq.) %B: 15–100 (50 min) 0.2 mL/min
*Palmaria spp.*, *Porphyra* spp., *Himanthalia elongata*, *Laminaria ochroleuca*, *Undaria pinnatifida* [201]	Sep. and detection of 7 phlorotannins	RP-HPLC-ESI-TOF	(-) FULL SCAN	Mediterranean Sea18 150 × 4 mm, 3 μm	A: 1% AcAc (aq.) B: 1% AcAc in ACN:water (32:68) %B: 0–100 (25 min) 0.5 mL/min
*Gracilaria birdiae*, *Gracilaria córnea* [193]	Detection and tentative ident. of 2 misc. phenolics	RP-HPLC-ESI-?	(+)/(-) MRM	Supelcosil LC-18 250 × 5 mm, -	A: MeOH B: 0.1% FA (aq.) %B: 50 (isocratic) 0.4 mL/min
*Ascophyllum nodosum* [77]	Sep. and ident. of 2 phlorotannins (DP-level)	RP-HPLC-ESI-IT	(+)/(-) FULL SCAN	Synergi Hydro C18 with polar end capping 150 × 2 mm, -	A: Water B: ACN %B: 5–100 (30 min) 0.2 mL/min
*Cystoseira nodicaulis*, *Cystoseira tamariscifolia*, *Cystoseira usneoides*, *Fucus spiralis* [35]	Sep. and ident. 22 of phlorotannins	RP-HPLC-ESI-IT	(+)/(-) FULL SCAN	Luna C18 250 × 4.6 mm, 5 µm	A: 1% FA (aq.) B: ACN %B: 0–80 (40), 80−0 (52) 1.0 mL/min
*Ascophyllum nodosum* [88]	Sep. and ident. of 6 phlorotannins (DP-level)	RP-HPLC-ESI-IT	(+)/(-) FULL SCAN/DDA	Synergi Hydro C18 w/polar end-capping 2.0 × 150 mm, -	A: 0.1% FA in ACN:water (5:95) B: 0.1% FA in ACN:water (40:60) %B: 0–100 (30 min) 0.2 mL/min
*Ecklonia bicyclis*, *Ecklonia kurome*, *Ecklonia arborea*, *Ecklonia cava* [202]	Sep. and ident. of 12 phlorotannins (DP-level)	RP-HPLC-ESI-Q	(-) FULL SCAN	Inertsil ODS-3 250 × 4.6 mm, 4 µm	A: water B: MeOH %B: 20–100 (40 min) -
*Ascophyllum nodosum*, *Laminaria digitata* [163]	Detection of phlorotannins	RP-HPLC-ESI-IT	(-) FULL SCAN	Nova-Pak C18 -	A: Formate:methanol 95:5 *v/v* B: MeOH %B: 0–100 (30 min) 1.0 mL/min
*Macrocystis pyrifera* [69,71]	Sep. and tentative ident. of 2 phlorotannins	RP-HPLC-ESI-IT	(+)/(-) FULL SCAN	LunaC18 150 × 4.6 mm, 5 μm	A: 1% FA (aq.) B: ACN %B: 5 (0–15 min), 5−100 (75–85 min), 100−5 (90 min) 1.0 mL/min
*Ascophyllum nodosum* [203]	Sep. and tentative ident. of 6 phlorotannins	RP-HPLC-ESI-IT	(-) FULL SCAN	Zorbax SB C18 100 × 2.1 mm, 1.8 µm	A: 0.1% FA (aq.) B: 0.1% FA in ACN %B: 10–70 (50), 70−10 (65) 2.0 mL/min
*Sargassum muticum* [96]	Detection and tentative identification of 3 phlorotanins.	RP-HPLC-ESI-IT	(+)/(-) FULL SCAN	Zorbax Eclipse XDB-C18 150 × 4.6 mm, 5 μm	A: 0.1% FA (aq.) B: ACN %B: 2–20 (35 min) 0.2 mL/min
*Ulva intestinales*, *Porphyra umbilicalis*, *Palmaria palmata*, *Fucus sechees*, *Himanthalia elongata*, *Laminaria japonica*, *Saccorhiza polyschides*, *Ascophyllum nodosum*, *Fucus vesiculosus*, *Laminaria digitata*, *Undaria pinnatifida* [146]	Sep. and detection of 22 phlorotannins	RP HPLC-ESI-QqTOF	(-) FULL SCAN	Luna C18 150 × 4.6 mm, 3 μm	A: 0.1% FA, 5% ACN (aq.) B: 0.1% FA in ACN:water (95:5) %B: 40–100 (65 min), 100−40 (67 min) 0.5 mL/min
*Himanthalia elongata* [17]	Sep. and ident. of 8 misc. phenolics	RP-HPLC-ESI-QqQ	(-) FULL SCAN	Atlantis C-18 250 × 4.6 mm, 5 μm C-18 guard cartridge 4.0 × 3.0 mm, -	A: 0.25% aqueous AcAc B: 0.25% AcAc in ACN:water (80/20) %B: 10 (20 min), 10−20 (30 min) 1.0 mL/min
*Achophyllum nodosum*, *Bifurcaria bifurcata*, *Fucus vesiculosus* [118]	Sep and ident. of 41 misc. phenolics	RP-HPLC-ESI-QqQ	(-) FULL SCAN	Zorbax SB C18 150 × 3 mm, 3.5 μm	A:2.5% AcAc (aq.) B: 2.5%AcAc in ACN %B: 5–15(0–15 min), 15−30 (35 min), 30−40 (40 min) –60 (50 min) − 90 (55 min) − 100 (55.01–75 min) 1.0 mL/min
*Dasycladus vermicularis* [21]	Sep and ident. of 4 coumarins and 2 phenolic acids	RP-HPLC-ESI-IT	(-) FULL SCAN	Gemini C18 150 × 4.6 mm, 3 μm	A: 20 mM ammonium acetate/1.5% AcAc (aq.) B: MeOH:water (90:10) %B: 2–15 (5 min), 15−60 (20 min), 60−98 (25 min) 0.3 mL/min
*Agarum cribrosum* [60]	Identification of 1 trifuhalol	RP HPLC-DAD-ESI-Q	(+) FULL SACN	Zorbax Analytical Rx-C18 250 × 4.6 mm, 5 μm	A: 0.1%FA (aq.) B: 0.1% FA in ACN %B: 5–15 (0–8 min), 15−30 (10 min), 30−35 (18 min), 35−100 (23–35 min), 100−5 (40 min) 1.0 mL/min
*Ecklonia arborea*, *Solieria filiformis* [119]	Sep and ident. of 1chromone, 5 flavonoid glycosides and 3 phlorotannins	RP-HPLC-ESI-TOF-	(+) FULL SCAN	SunFire C18 150 × 4.6 mm, 5 μm	A: 3% AcAc (aq.) B: 3% AcAc in ACN %B: 10 (0–3.5 min), 10−50 (5 min), 50−0 (10–20 min) 1.5 mL/min
*Ascophyllum nodosum*, *Laminaria japonica*, *Lessonia trabeculate*, *Lessonia nigrecens* [110]	Sep., ident. and quant. of 12 misc. phenolics	RP-HPLC-ESI-Q	(-) FULL SCAN	Hypersil Gold C-18 150 × 2.1 mm	A: 0.25% AcAc (aq.) B: CAN:water(80:20) %B: 40 (20 min), 40−75 (35 min), 75−90 (46 min), 90−40 (50 min) 0.2 mL/min
*Cladophora glomerata* [14]	Sep., ident. and quant. of 10 phenolic acids and flavonoids	RP-HPLC-ESI-IT	(-) MRM	LichroCART C-18 150 × 4.6 mm, 5 µm	A: 10mM ammonium formate/FA (pH4) B: ACN %B: 0 (0–5 min). 0−20 (10 min), 20 (20 min), 20−40 (60 min) 1.0 mL/min
*Himanthalia elongata* [64]	Sep, ident. and quant. of 7 phenolic acids and flavonoids	RP-HPLC-ESI-QqQ	(-) FULL SCAN	Atlantis C-18 250 × 4.6 mm, 5 µm	A: 0.25% AcAc (aq.) B: 0.25%AcAc in ACN:water(80:20) %B: 10 (0–20 min), 10−20 (30 min), 20−30 (35 min), 30−0(45 min) 1.0 mL/min
*Fucus guiryi*, *Fucus serratus*, *Fucus spiralis*, *Fucus vesiculosus* [58]	Sep and ident. of 22 phlorotannins (DP-level)	RP-HPLC-ESI-IT	(-) FULL SCAN	Kinetex 150 × 4.6 mm, 5 µm	A: 1% FA (aq.) B: ACN %B: 1–10 (12 min), 10−30 (25 min), 30−50 (27 min), 50−1 (29 min) 0.8 mL/min
		RP-UHPLC-ESI-QTOF	(-) FULL SCAN	Luna Omega 50 × 2.1 mm, 1.6 µm	A: 0.1% FA(aq.) B: 0.1% FA in ACN %B: 1–10 (8 min), 10−30 (14 min), 30−50 (16 min), 50–1 (20 min) 0.5 mL/min
*Ecklonia cava* [116]	Sep and ident. of 15 phlorotannins	RP-HPLC-ESI-qTOF	(+)/(-) FULL SCAN	YMC-Triart C18 150 × 3.0 mm, 5 µm	A: 0.01% FA (aq.) B: 0.01% FA in ACN %B: 10–60 (40 min), 60 (45 min)/10–100 (40 min), 100(45 min) 0.3 mL/min
*Ulva intestinalis*, *Dictyota dichotoma* [57]	Sep and ident. of 1 flavone	RP-HPLC-ESI-TOF	(+)/(-) FULL SCAN	Zorbax SB-C18 100 × 2.1 mm, 3.5 μm	A: 0.05 FA (aq.) B:ACN %B: 10–90 (60 min), 90−10 (65 min) 0.3 mL/min
		RP-HPLC-ESI-Q	(-) SIM	Zorbax SB-C18 100 × 2.1 mm, 3.5 μm	A: 0.05 FA (aq.) B:ACN %B: 10–90 (90 min), 90−10 (95 min) 0.3 mL/min
*Sargassum pallidum* [195]	Sep. and ident. of 6 phlorotannins (DP-level)	RP-UHPLC-ESI-QqQ	(-) FULL SCAN	Bridged Ethyl Hybrid (BEH) C18 100 × 2.1 mm, 1.7 μm	A: MeOH B: water %B: 30–100 (10 min),100–30 (20 min) 0.3 mL/min
*Ecklonia cava* [152]	Sep. and ident. of 4 phlorotannins	RP-UHPLC-ESI-?	(+) FULL SCAN	Bridged Ethyl Hybrid (BEH) C18 100 × 2.1 mm, 1.7 μm	A: 0.1% FA (aq.) B: 0.1% FA in ACN %B: 10–45 (5 min) 0.4 mL/min
*Ascophyllum nodosum*, *Pelvetia canaliculata*, *Fucus spiralis* [165]	Sep. and ident. of >100 phlorotannins (DP-level, isomers)	RP-UHPLC-ESI-QqQ	(-) MRM	HSS PFP 100 × 2.1 mm, 1.8 μm	A: 0.1% FA (aq.) B: 0.1% FA in ACN %B: 0.5–90 (28 min), 90−0.5 (30 min) 0.5 mL/min
*Fucus serratus*, *Fucus vesiculosus*, *Himanthalia elongata*, *Cystoseira nodicaulis* [28]	Sep. and ident. of >100 phlorotannins (DP-level, isomers)	RP-UHPLC-ESI-QqQ	(-) MRM	HSS PFP 100 × 2.1 mm, 1.8 μm	A: 0.1% FA (aq.) B: 0.1% FA in ACN %B: 0.5–90 (28 min), 90−0.5 (30 min) 0.5 mL/min
*Fucus vesiculosus* [166]	Sep. and ident. of >100 phlorotannins (DP-level, isomers)	RP-UHPLC-ESI-QqQ	(-) MRM	HSS PFP 100 × 2.1 mm, 1.8 μm	A: 0.1% FA (aq.) B: 0.1% FA in ACN %B: 0.5–90 (28 min), 90−0.5 (30 min) -
*Laminaria digitata* [44]	Sep. and ident. of 34 phlorotannins (Class and DP-level)	RP-UHPLC-ESI-IT	(-) FULL SCAN	Bridged Ethyl Hybid (BEH) C18 150 × 2.1 mm, 1.7 μm	A: 0.1% FA (aq.) B: 0.1% FA in ACN %B: 1 (1 min), 1−8 (1.5 min), 8−55 (24 min), 55−100 (25–28 min), 100−1 (29 min) 0.4 mL/min
*Fucus vesiculosus* [59]	Sep and ident. of 13 phlorotannins (Class and DP-level)	RP-UHPLC-QTOF	(+)/(-) FULL SCAN	Prodigy ODS 3 150 × 2 mm, 3 μm	A: Ammonium formate buffer/20 mM FA, pH3 B: 20 mM FA in ACN %B: 0 (0–2 min), 0−40 (16 min), 40−100 (18 min)
*Fucus vesiculosus* [106]	Sep and ident. of 1 phlorotannin	RP-UHPLC-ESI-QTOF-MS	(+) FULL SCAN, DDA	HSS T3 100 × 2.1 mm, -	A: 0.1% FA (aq.) B: ACN %B 1–40 (2.5 min), 40−100 (13.5–14.5 min), 100−1 (14.6 min) 0.6 mL/min
*Pelvetia canaliculata*, *Fucus spiralis*, *Fucus vesiculosus*, *Ascophyllum nodosum*, *Saccharina longicruris* [10]	Sep. and ident. of 21 phlorotannins (DP-level)	HILIC-UHPLC-ESI-Orbitrap	(-) FULL SCAN	UHPLC W BEH Amide 100 × 2.1 mm, 1.7 μm	A: 10.0mM ammonium acetate pH 9.0 B: ACN. %B: 95–65 (16 min), 95 (21 min) 0.4 mL/min
*Enteromorpha prolifera* [120]	Sep and ident. of 1 naphtalenone, 1 flavone and 1 flavanone	RP-UHPLC-ESI-QuanTOF	(+) FULL SCAN	Unspecified C18 100 × 2.1 mm, 1.7 µm	A: 0.1% FA (aq.) B: 0.1% FA in ACN %B: 1 (0–0.25 min), 1−99 (16.25 min), 99 (17 min) 0.45 mL/min
*Fucus vesiculosus* [9]	Sep. and tentative ident. of 17 phlorotannins	RP-UHPLC-ESI-IT	(-) FULL SCAN	Hypersil Gold-C18 100 × 2.1 mm, 1.9 µm	A: 0.1 % FA (aq.) B: ACN %B: 5–40% (14.72 min), 40–100% (1.91 min), 100 (2.19 min). 0.2 mL min^−1^
*Durvillaea antarctica*, *Lessonia spicata*, *Macrocystis integrifolia* [19]	Sep and ident. of 84 miscellaneous phenolics	RP-HPLC-ESI-IT/n		Unspecified C18 250 × 4.6 mm, 5 µm	A: 1% FA (aq.) B: ACN %B: 5 (0–5 min), 5−30 (60 min), 30−60 (70–80 min), 60−5 (90 min) 1.0 mL/min
*Cystoseira abies-marina* [36]	Sep. [52 + spots] and ident. of phlorotannins (DP-level)	HILICxRP-2D-HPLC-ESI-IT	(-) FULL SCAN	D1 precolumn Lichrospher diol-5 Lichrospher diol-5 150 × 1.0 mm, 5 µm D2 precolumn C18 Ascentis Express C18 50 ×4.6 mm, 2.7 μm OR Kinetex pentafluorophenyl (PFP) 50 × 4.6 mm, 2.6 μm	D1 A: 2% AcAc in ACN B: 2% AcAc, 3% water in MeOH %B: 0–25 (85 min) 15 μL/min D2 A: 0.1% FA (aq.) B: ACN %B: 0–90 (0.9 min), 90−0( 1.3 min) 3 mL/min
*Sargassum muticum* [104]	Sep. [73+ spots] and tentative ident. of phlorotannins (Class-level)	HILICxRP-2D-HPLC-ESI-IT	(-) FULL SCAN	D1 Lichrospher diol-5 150 × 1.0 mm, 5 μm D2 C18 precolumn Ascentis Express C18 50 × 4.6 mm, 2.7 μm	D1 A: 2% AcAc in ACN B: 2% AcAc, 3% water in MeOH %B: 0–25 (85 min) 15 μL/min D2 A: 0.1% FA (aq.) B: ACN %B: 0–90 (0.9 min) − 0 (1.3 min) 3 mL/min
*Cystoseira abies-marina* [194]	Sep. [35 spots] and ident. of 29 phlorotannins (DP-level)	HILICxRP-2D-HPLC-ESI-IT	(-) FULL SCAN	D1 precolumn Lichrospher diol-5 Lichrospher diol-5 150 × 1.0 mm, 5 µm D2 C18 precolumn Partially porous C18 50 × 4.6 mm, 2.7 µm	D1 A: ACN/AcAc (98:2, *v/v*) B: MeOH/water/AcAc (95:3:2, *v/v/v*) %B: 3–35 (70 min) 15 µL/min D2 A: 0.1% FA (aq.) B: ACN %B: 0–90 (1 min), 90−0 (1.01 min), − repeat 3 mL min^−1^

^a^ NP—normal phase; HPLC—high performance liquid chromatography, ESI—electrospray ionization; QqQ—triple quadrupole; SEC—size exclusion chromatography; IT—ion trap; TOF—time of flight; RP—reversed phase; Q—single quadrupole; IT/n—ion trap with tandem spectrometry acquisition; Qq—quadrupole-quadrupole; UHPLC—ultra high-performance liquid chromatography; QuanTOF—quantitative TOF; HILIC—hidrophilic interaction; 2D—two dimensions. ^b^ SIM—selection ion monitoring; MRM—multiple reaction monitoring; DDA—data-dependent acquisition. ^c^ DCM—dichloromethane; MeOH—methanol; ACN—acetonitrile; AcAc—acetic acid; FA—formic acid; TFA—trifluoroacetic acid; aq.—aqueous.

**Table 10:** Compiled MS data on the phlorotannins with DP = 1 and DP = 2.

DP	Class	Compound *IUPAC Name*	Formula	Mass Spectrometry	Reference
**1**	Phloroglucinol	Phloroglucinol 1,3,5-trihydroxybenzene	C_6_H_6_O_3_	*Native* EI: 126 ([M]^+^, 100), 111 (9), 97 (20), 85 (30), 69 (41), 52 (26) ESI: 125.05 ([M-H]^−^, 100), 97.0 (-), 80.0 (-)	[17,153,186]
	Phloroglucinol (H)	2-Bromo-phloroglucinol 2-bromo-1,3,5-trihydroxybenzene	C_6_H_6_BrO_3_	*Native* EI: 206/204 ([M]^+^, 100/91), 188/186 (4.7/5.3), 177/175 (4.2/4.0), 97 (19), 85 (11), 79 (11), 78 (14), 69 (88) *Tri-Ac* EI: 332/330 ([M]^+^, -/-), 290/288 (-/-), 248/246 (-/-), 206/204 (-/-)	[45,213]
		2-Chloro-phloroglucinol 2-chloro-1,3,5-trihydroxybenzene	C_6_H_6_ClO_3_	*Tri-Ac* EI: 288/286 ([M]^+^, -/-), 246/244 (-/-), 204/202 (-/-), 162/160 (-/-)	[40]
		2-Iodo-phloroglucinol 2-iodo-1,3,5-trihydroxybenzene	C_6_H_6I_O_3_	*Tri-Ac* EI: 378 (-), 336 (-), 294 (-), 252 (-), 210 (-), 168 (-), 126 (-)	[45]
**2**	Fucol	Difucol 2,2′,4,4′,6,6′-Hexahydroxybiphenyl	C_12_H_10_O_6_	*Hexa-Met* EI: 334 (100), 181 (9.5) *Hex-Ac* EI: 502 ([M]+, -), 460 (-), 418 (-), 376 (-), 334 (-), 292 (-), 250 (-)	[208,209]
		Difucol-4,4′-disulphate (dipotassium salt) 2,2′,6,6′-Tetrahydroxybiphenyl-4,4′-disulphate	C_12_H_10_S_2_O_12_	*Native* FAB: 485 ([M]^+^, 25), 447 (100),409 (15), 367 (30), 351 (40), 329 (50), 281 (35), 243 (100), 227 (35), 205 (15) *Tetra-Ac* FAB: 615 ([M]^+^, 70), 599 (17), 577 (15), 573 (40), 557 (5), 535/531 (20), 497 (25), 493 (15), 481 (4), 469 (12), 455 (20), 289 (100), 273 (31), 255 (31), 249 (56), 243 (51), 230 (31), 215 (51)	[39,214]
	Fucol (H)	3-Chloro-difucol 3[A4]-Chloro-difucol 3-Chloro-2,2′,4,4′,6,6′-biphenylhexol	C_12_H_9_ClO_6_	*Hex-Ac* FAB: 559 ([M+Na]^+^, -), 537 ([M+H]^+^, -), 495 (-), 453 (-), 411 (-), 369 (-), 327 (-), 285 (-) EI: 538/536 ([M]^+^, -/-), 496/494 (-/-), 454/452 (-/-), 412/410 (-/-),…,370/368 (-/-), 328/326 (-/-), 286/284 (-/-)	[40]
	Fuhalol	Bifuhalol 5-(2,4,6-Trihydroxyphenoxy)-1,2,3-benzenetriol	C_12_H_10_O_7_	*Native* ESI: 265 ([M-H]^-^, -), 247 (-), 141 (-), 139 (-), 125 (-), 123 (-), 111 (-) *Hex-Ac* EI: 518 (-), 476 (-), 434 (-), 392 (-), 350(-), 308 (-), 266 (-), 142 (-), 126 (-)	[210,211]
	Fuhalol (H)	3′-Chloro-bifuhalol 3[A]-Chloro-bifuhalol 5-(3-chloro-2,4,6-Trihydroxyphenoxy)-benzene-1,2,3-triol	C_12_H_9_ClO_7_	*Hex-Ac* FAB: 591 ([M+K]^+^, -) 575 ([M+Na]^+^, -), 553 ([M+H]^+^, -), 511 (-), 469 (-), 427 (-), 385 (-), 343 (-), 301 (-) EI: 554/552 (-/-), 512/510 (-/-), 470/468 (-/-), 428/426 (-/-), 386/384 (-/-), 344/342 (-/-), 302/300 (-/-)	[40]
	Phlorethol	Diphlorethol 2-(3,5-dihydroxyphenoxy)benzene-1,3,5-triol	C_12_H_10_O_6_	*Native* ESI: 251 ([M+H]^+^, -), 233 (-), 139 (-), 123 (-), 109 (-), 93 (-) *Penta-Ac* EI: 460 ([M]^+^, -), 418 (-), 376 (-), 334 (-), 292 (-), 250 (-), 142 (-), 126 (-), 110 (-)	[210,212]
	Phlorethol (H)	3-Bromo-diphlorethol 3[A]-Bromo-diphlorethol 3[A1]-Bromo-diphlorethol 2-(3,5-Dihydroxyphenoxy)-3-bromo-1,5-benzenediol	C_12_H_9_BrO_6_	*Penta-Ac* EI: 540/538 ([M]+, 3/3), 498/496 (21/20), 456/454 (40/40), 414/412 (65/65), 372/370 (45/44), 330/328 (28/29), 418 (1), 376 (1), 334 (3), 292 (6), 250 (2), 248 (12), 231 (9), 69 (18), 43 (100)	[41,46]
		2′-Bromo-diphlorethol 2[D]-Bromo-diphlorethol 2[D′]-Bromo-diphlorethol 2-(2′-bromo-3′,5′-Dihydroxyphenoxy)-1,3,5-benzenetriol	C_12_H_9_BrO_6_	*Pent-Ac* EI: 540/538 ([M]+, 3/3), 498/496 (21/20), 456/454 (40/40), 414/412 (65/65), 372/370 (45/44), 330/328 (28/29), 418 (1), 376 (1), 334 (3), 292 (6), 250 (2), 248 (12), 231 (9), 69 (18), 43 (100)	[41,46]
		4′-Bromo-diphlorethol 4[D]-Bromo-diphlorethol 4[D′]-Bromo-diphlorethol 2-(4-bromo-3,5-Dihydroxyphenoxy)-1,3,5-benzenetriol	C_12_H_9_BrO_6_	*Pent-Ac* EI: 540/538 ([M]+, 3/3), 498/496 (21/20), 456/454 (40/40), 414/412 (65/65), 372/370 (45/44), 330/328 (28/29), 418 (1), 376 (1), 334 (3), 292 (6), 250 (2), 248 (12), 231 (9), 69 (18), 43 (100)	[41,46]
	Phlorethol (H)(Cont.)	4′-Chloro-diphlorethol 4[D]-Chloro-diphlorethol 4[D′]-Chloro-diphlorethol 2-(4-bromo-3,5-Dihydroxyphenoxy)-1,3,5-benzenetriol	C_12_H_9_ClO_6_	*Pent-Ac* EI: 496/494 (-/-), 454/452 (-/-), 412/410 (-/-), 370/368 (-/-), 328/326 (-/-), 286/284 (-/-)	[41]
		2′-Iodo-diphlorethol 2[D′]-Iodo-diphlorethol 2′-iodo-2-(3,5-Dihydroxyphenoxy)-1,3,5-benzenetriol	C_12_H_9_IO_6_	*Pent-Ac* EI: 586 (-), 544 (-), 502 (-), 460 (-), 418 (-), 376 (-)	[40]
